# Order–Disorder Phase Stabilization by Pressure‐Induced Charge Transfer Enhances the Ferroelectric Photovoltaic Effect in Multiferroic BaFe_4_O_7_

**DOI:** 10.1002/advs.202511022

**Published:** 2025-08-19

**Authors:** Jiayi Guan, Bihan Wang, Nana Li, Shang Peng, Ganghua Zhang, Limin Yan, Xuqiang Liu, Kai Zhang, Mingtao Li, Adama N‐Diaye, Qingyu Kong, Dongzhou Zhang, Xu Zhao, Ting Liu, Kejun Bu, Yuhong Mao, Gui Wang, Xujie Lü, Xiang Li, Tao Zeng, Wenge Yang

**Affiliations:** ^1^ School of Physics Beijing Institute of Technology Beijing 100081 P. R. China; ^2^ Center for High Pressure Science and Technology Advanced Research (HPSTAR) Shanghai 201203 P. R. China; ^3^ School of Materials Science and Engineering Shanghai Institute of Technology Shanghai 201418 P. R. China; ^4^ Synchrotron SOLEIL L'Orme des Merisiers Saint‐Aubin‐BP48 Gif‐sur‐Yvette Cedex 91192 France; ^5^ GSECARS Advanced Photon Source University of Chicago 9700 S Cass Avenue Chicago IL 60439 USA; ^6^ Shanghai Key Laboratory of Engineering Materials Application and Evaluation Shanghai Research Institute of Materials Shanghai 200437 P. R. China; ^7^ National Engineering Research Center for Domestic and Building Ceramics Jingdezhen Ceramic University Jingdezhen 333000 China; ^8^ Deutsches Elektronen‐Synchrotron DESY Notkestr. 85 22607 Hamburg Germany

**Keywords:** bandgap engineering, ferroelectric photovoltaic effect, high pressures, phase transitions

## Abstract

Multiferroic ferroelectric photovoltaic (FPV) materials, combining magnetic and ferroelectric properties, are of paramount importance for optoelectronic and photovoltaic applications. However, optimizing both the remanent polarization and the optical bandgap—key factors for enhanced FPV performance—presents a significant challenge due to their trade‐off. This work shows that pressure‐induced charge transfer between different metal sites can break this trade‐off. Above ≈20 GPa, charge transfer between different trivalent iron (Fe) sites in the multiferroic material BaFe_4_O_7_ leads to Fe valence disproportionation, FeO_4_ tetrahedra disorder, and Jahn–Teller distortion of FeO_6_ octahedra. These changes reduce the bandgap, lower resistivity, and enhance ferroelectric polarization, resulting in a 2.5‐fold increase in photocurrent. Upon decompression, BaFe_4_O_7_ retains an order–disorder structure, optimal ferroelectric and optical properties at ambient conditions. This work provides a novel pathway to simultaneously optimizing ferroelectricity and bandgap via pressure‐induced charge transfer, overcoming the traditional trade‐off in FPV materials, and offers a promising approach for developing high polarization performance, narrow‐bandgap FPV materials.

## Graphical abstract

A pressure‐driven charge transfer in multiferroic BaFe4O_7_ induces Fe valence disproportionation and structural distortion, drastically narrowing the bandgap and enhancing ferroelectric polarization. This synergistic optimization boosts photocurrent 2.5‐fold and is retained upon decompression, offering a promising strategy to overcome the ferroelectric–bandgap trade‐off in high‐performance ferroelectric photovoltaic materials.

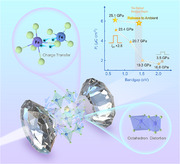

## 1 Introduction

Multiferroic materials have garnered significant attention due to their coupled electric, magnetic, and structural order parameters, making them ideal for multifunctional devices. Among them, the ferroelectric photovoltaics (FPV) effect offers an alternative method for solar energy conversion, surpassing the Shockley–Queisser limit through intrinsic polarization‐driven charge separation.^[^ ^1^, ^2^ ^]^ However, practical efficiency is limited by weak light absorption, poor charge mobility, and insufficient control over the internal polarization field. Traditional FPV materials often suffer from large bandgaps and low conductivity, hindering their broad application.^[^ ^3^, ^4^, ^5^ ^]^ To enhance FPV performance, two key strategies are critical: boosting ferroelectric polarization to improve the separation of photogenerated carriers and optimizing the bandgap to increase light absorption to increase the photogenerated carriers. Extensive studies have been focused on reducing the bandgap of ferroelectric materials using various physical and chemical methods such as doping,^[^ ^6^, ^7^ ^]^ strain,^[^ ^8^, ^9^ ^]^ and thickness adjustments.^[^ ^10^, ^11^ ^]^ However, these approaches often lead to high leakage currents, which affect the screening charges and potentially eliminate the separation field, thereby weakening the intrinsic ferroelectric polarity.^[^ ^12^ ^]^ For instance, (1 −* x*)K_0.5_Na_0.5_NbO_3‐x_Sr(In_0.5_Ta_0.5_)O_3_ transparent ferroelectric ceramics exhibit improved optical properties as the Sr(In_0.5_Ta_0.5_)O_3_ content increases, but their ferroelectric properties degrade.^[^ ^13^ ^]^ As a result, achieving simultaneous improvements in both polarization and bandgap has become a key focus for optimizing the FPV performance. To improve photocurrent, it is crucial to effectively separate the photogenerated carriers in FPV. In the ballistic and shift current mechanism of FPV,^[^ ^14^, ^15^ ^]^ carrier separation is driven by the coherent evolution of electron and hole wavefunctions, rather than by the built‐in electric field. Photocurrents can be generated in bulk when scattering sites or absorption centers exhibit some degree of asymmetry. The ballistic current reflects asymmetric electron excitation in momentum space and is influenced by the depolarization field. Thus, a promising approach to enhancing photocurrent should incorporate two key elements: increasing lattice distortion or symmetrical breaking to enhance ferroelectric properties and optimizing the bandgap for better light absorption and efficient transport of photogenerated carriers to enhance photocurrent. Given the above insights, there is a strong impetus to develop new strategies that effectively combine the benefits of narrow‐bandgap semiconductors with ferroelectric materials.

Pressure, as a precise tuning tool, can significantly influence the interplay between lattice, charge, spin, and orbital interactions in multiferroic materials.^[^ ^16^, ^17^, ^18^ ^]^ Over the last decades, pressure‐induced structural and electronic evolutions have boosted profound research attention, including but not limited to lattice distortion, intermetallic charge transfer, spin‐crossover, etc. These changes can significantly influence ferroelectric polarization,^[^ ^19^ ^]^ electrical properties,^[^ ^20^ ^]^ and magnetic properties.^[^ ^21^ ^]^ For instance, in LaCu_3_Fe_4_O_12_, pressure‐induced charge transfer from Cu to Fe (LaCu^3+^_3_Fe^3+^_4_O_12_ to LaCu^2+^_3_Fe^3.75+^_4_O_12_ at 3.6 GPa) results in transitions from insulator‐to‐metal and antiferromagnetism‐to‐paramagnetism.^[^ ^22^ ^]^ Similarly, pressure‐induced charge transfer between Ag and Ru in AgRuO_3_ reveals successive insulator—metal–insulator transitions.^[^ ^23^ ^]^ In the PbCoO_3_ system, charge transfer between Pb^4+^ and Co^2+^ under high pressure induces a series of transitions in PbCoO_3_, including a spin state transition of Co^2+^, a metal‐insulator transition, and reentrant insulating behavior.^[^ ^21^ ^]^ The mixed valence resulting from intermetallic charge transfer under pressure in Ba_3_BiM_2_O_9_ (*M* = Ru, Ir) and Cu_2_IrO_3_ highlights the role of *d*‐orbital spatial distribution in shaping their electronic properties.^[^ ^24^, ^25^ ^]^ Furthermore, pressure‐induced changes from a charge disproportionated state (Bi^3+^_0.5_Bi^5+^_0.5_Ni^2+^O_3_) to a metallic charge melting state (Bi^3+^Ni^3+^O_3_) in BiNiO_3_, with notable charge transfer from Ni to Bi and a broad temperature invar behavior.^20^ These examples underscore the sensitivity of transition metal oxide electrical properties to internal metallic charge exchange. Thus, pressure‐induced intermetallic charge transfer in multiferroic materials offers promising potential for reducing the bandgap and enhancing electrical conductivity. However, semiconductor‐to‐metal transitions are frequently observed in materials undergoing pressure‐induced charge transfer, which can impair the ability of ferroelectric materials to maintain high polarization.

**Figure 1:**
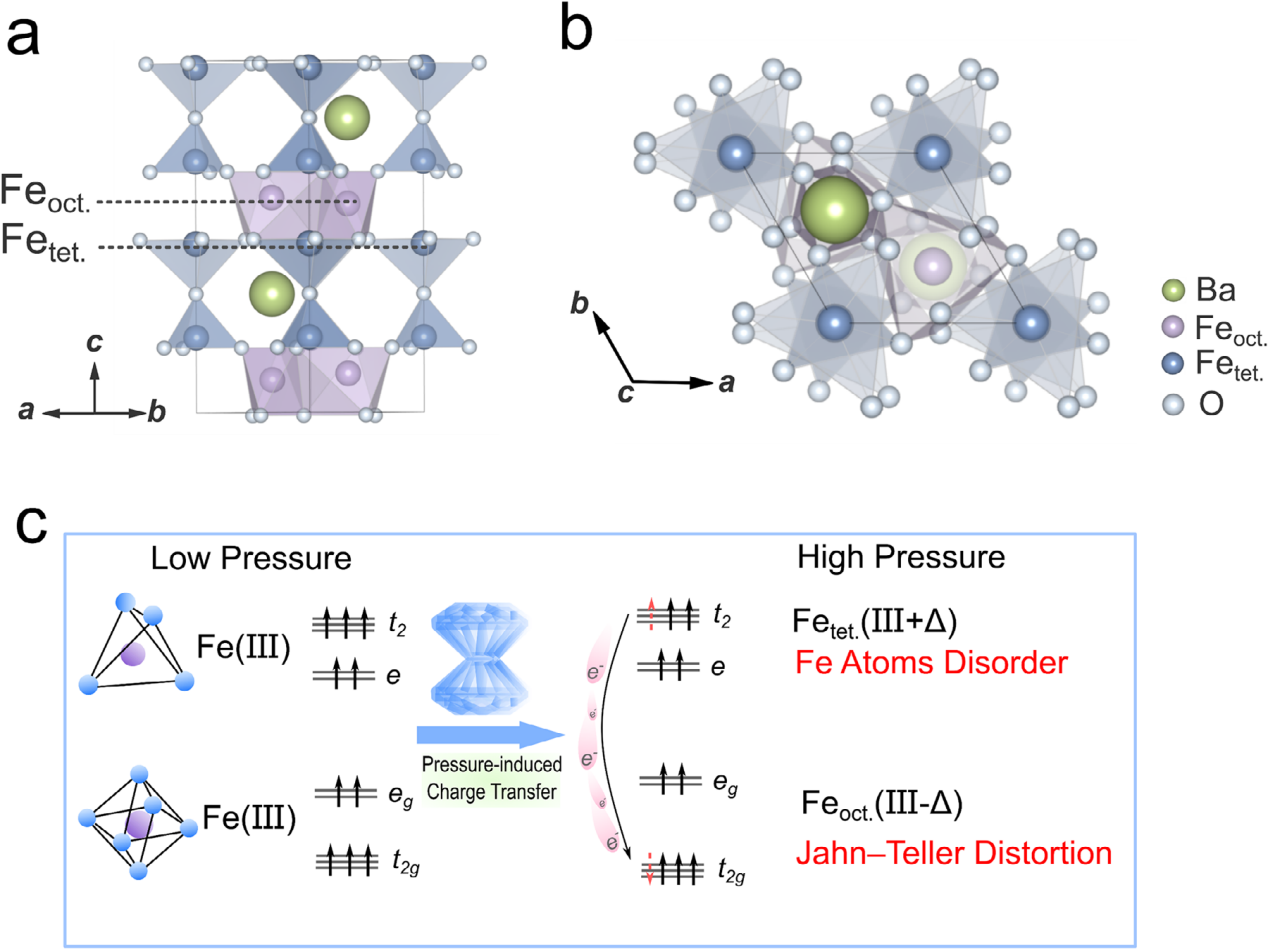
Crystal structure at ambient conditions, optimized ferroelectric photovoltaic mechanism, and detection method of BaFe_4_O_7_. a,b)The unique FeO_4_ tetrahedral and FeO_6_ octahedral interleaving arrangement of BaFe_4_O_7_ at ambient condition. Fe_oct._ refers to the Fe atom at the octahedral site, and Fe_tet._ refers to the Fe atom at the tetrahedral site. c) Schematic diagram of the optimized ferroelectric photovoltaic mechanism in this work.

In ordered–disordered systems, the disordered regions help sustain semiconducting behavior by introducing scattering centers that impede electron transport, thereby increasing resistance. Simultaneously, the ordered regions contribute to the structure stability and overall material properties. In this work, we investigate BaFe_4_O_7_,^[^ ^31^ ^]^ a multiferroic material featuring dual‐site high‐valence Fe^3+^ ions, to induce charge transfer under applied pressure, resulting in Fe valence disproportionation. Fe ions with oxidation states greater than +3 are inherently metastable; and this metastability mitigates long‐range atomic disorder caused by electronic instability, thereby preserving semiconducting properties. Conversely, Fe–O polyhedra with oxidation states lower than +3 experience an increased electron density, potentially triggering Jahn–Teller distortions that further enhance the material's ferroelectric polarization.^[^ ^26^, ^27^, ^28^ ^]^ (See **Figure** **1**c for a schematic diagram). BaFe_4_O_7_ exhibits intriguing ferroelectric photovoltaic, ferrimagnetic, and medium bandgap semiconducting properties at ambient pressure.^[^ ^29^ ^]^ The BaFe_4_O_7_ compound features a unique noncentrosymmetrical *P*3_1_ *c* structure, characterized by the interlacing of FeO_4_ tetrahedra and FeO_6_ octahedra with the transition metal ions (Fe^3+^) in different coordination environments, resulting in a medium bandgap of 2.12 eV at ambient conditions (as shown in Figure 1a,b). Under compression, distinct responses from iron ions at different sites are expected, providing significant tuning space for both the crystal and electronic structures. To this end, we explored charger transfer using high energy resolution X‐ray absorption spectroscopy (XAS), charge transfer induced order–disorder structural and FeO_6_ distortion using powder and single crystal X‐ray diffraction (P‐XRD and SC‐XRD) under high pressure. Additionally, in situ high‐pressure UV–vis–NIR absorption spectra, polarization–electric field (*P–E*) hysteresis loops, photocurrent measurements, low‐temperature resistance, and electrochemical impedance spectroscopy (EIS) are utilized to characterize the performances of electrical transport, optical absorption properties, ferroelectricity, and ferroelectric photovoltaic behavior under high pressure. Remarkably, upon decompression to ambient conditions, we were able to preserve the order–disorder structure and enhanced properties, offering an effective route to optimize the FPV properties for practical applications at ambient conditions.

## 2 Results

### 2.1 Pressure‐Induced Fe(III) Charge Transfer

**Figure 2:**
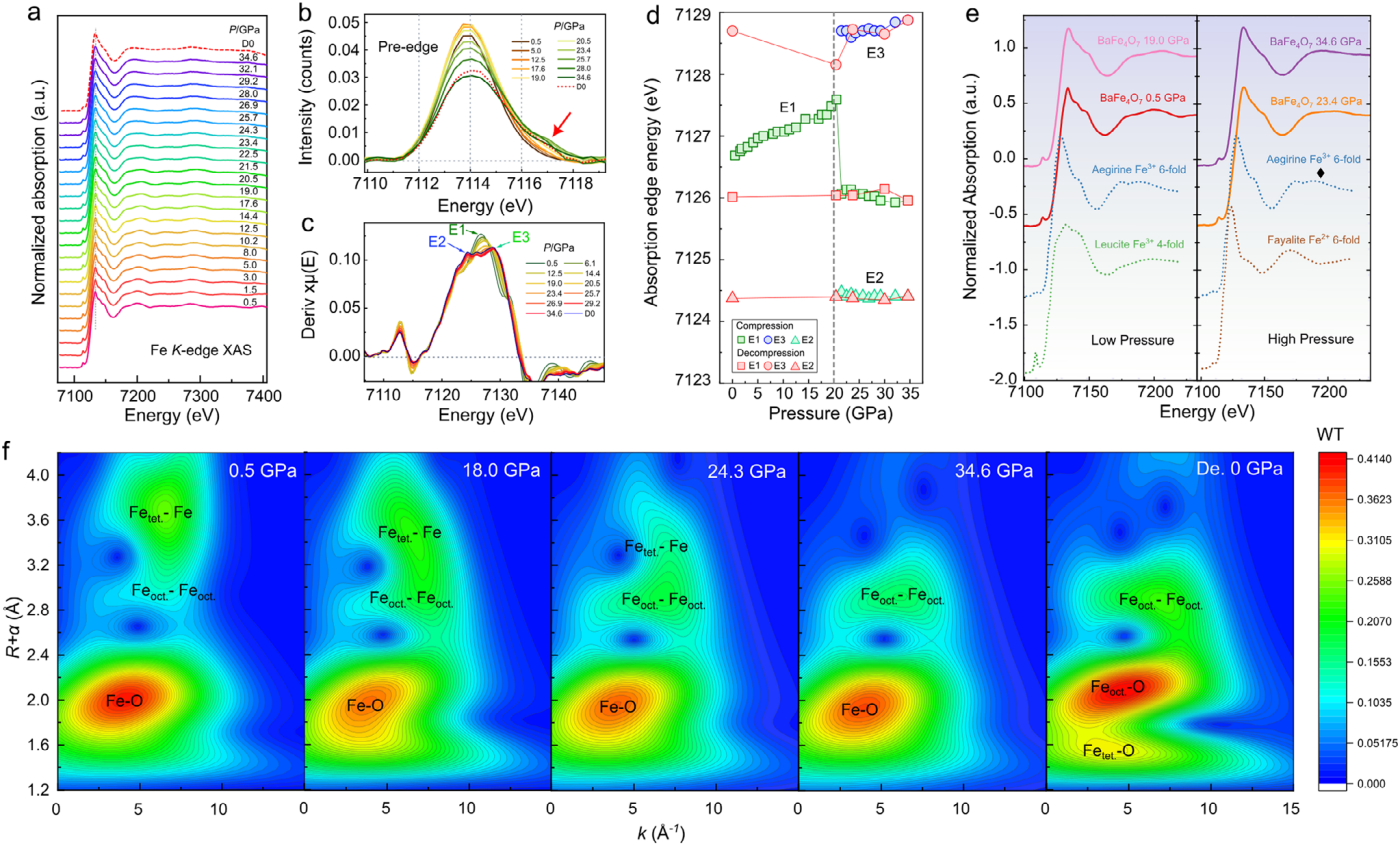
Fe *K*‐edge X‐ray absorption spectra (XAS) of BaFe_4_O_7_ under pressure. a) Fe *K*‐edge XAS at selected pressures. b) The pre‐edge signals at selected pressures. c) The derivative of XAS under various pressures. d) Pressure dependence of the absorption edge energy. e) The relationship between model mineral and BaFe_4_O_7_ under high pressure. f) The EXAFS‐WT (wavelet transformation) maps of BaFe_4_O_7_ at selected pressures.

To probe the effect of pressure on the electronic configuration of BaFe_4_O_7_, we conducted the high‐energy resolution X‐ray absorption spectroscopy (XAS) at Fe *K*‐edge at pressure up to 34.6 GPa, as shown in **Figure** **2**a. The pre‐edge peak features the *1s*→*3d* electronic transition, while the shift of the absorption edge (the maximum value of the first derivative of XAS) typically shifts due to changes in the oxidation state.^[^ ^30^, ^31^ ^]^ Figure 2b illustrates the pre‐edge feature of XAS at various pressures. A shift of 2–3 eV of the pre‐edge and absorption edge to higher energy at the Fe *K*‐edge is generally associated with one valence increase of Fe.^[^ ^32^, ^33^ ^]^ The Fe^2+^ pre‐edge is centered ≈7112 eV, while the Fe^3+^ pre‐edge is ≈7114 eV. In BaFe_4_O_7_, Fe^3+^ ions are located in both tetrahedral and octahedral coordination environments, which results in a strong pre‐edge peak ≈7114 eV. The pre‐edge feature of tetrahedral Fe^3+^ is nearly seven times stronger than that of octahedral Fe^3+^ due to *3d*‐*4p* orbital hybridization and *3d*‐ligand *2p*‐orbital overlap in a noncentrosymmetric coordination, which relaxes the forbidden character of the electric dipole transition and is further enhanced by site distortion.^[^ ^34^ ^]^ When pressure exceeds 20 GPa, the intensity of the pre‐edge peak significantly reduces, indicating the destruction of the Fe─O tetrahedra. Simultaneously, a small shoulder peak appears ≈7117 eV, indicating the emergence of a small portion of Fe^3+Δ^ oxidation state. The first‐order derivatives of XASs are plotted in Figure 2c. At 0.5 GPa, the peak value E1 of the first derivative corresponds to the Fe^3+^ absorption edge. At 21.5 GPa, this peak clearly splits into three peaks with a notable decrease in the intensity of E1, indicating the presence of mixed valence states. The newly emerging peaks are labeled as E2 and E3. The energies E1, E2, and E3 as a function of pressure are plotted together in Figure 2d. The energy of the E1 peak increases slightly with pressure below 21.5 GPa, which can be attributed to the contraction of the Fe─O bonds with pressure.^[^ ^35^ ^]^ The energy of the E2 peak that appears at 21 GPa has ≈2 eV lower than that of E1, while E3 has ≈3 eV notably higher than E1. This suggests that, above 21.5 GPa, Fe in BaFe_4_O_7_ adopts both lower and higher oxidation states than Fe^3+^, and these mixed states persist throughout the pressure range from 21.5 to 34.6 GPa and during the subsequent decompression. This provides direct evidence of intermetallic charge transfer occurring in BaFe_4_O_7_ above ≈20 GPa. In Figure 2e, we compare the high‐pressure XAS spectra of BaFe_4_O_7_ with those of several representative model minerals with sixfold and fourfold coordinated Fe^3+^ and Fe^2+^ (Aegirine with sixfold coordination Fe^3+^, Leucite with fourfold coordination Fe^3+^, Fayalite with sixfold coordination Fe^2+^)^[^ ^36^ ^]^ to further elucidate intermetallic charge transfer phenomena through the characteristic fingerprint effect of X‐ray absorption near‐edge structure (XANES). Since, under low pressure (below ≈20 GPa), the ratio of sixfold coordinate Fe^3+^ to fourfold coordinate Fe^3+^ in BaFe_4_O_7_ is 1:1, the XAS shape of BaFe_4_O_7_ resembles a superposed XANES spectrum of Aegirine and Leucite. Under high pressure (above 20 GPa), due to the disruption of the fourfold coordinate Fe^3+^, the XANES of BaFe_4_O_7_ under 23.4 GPa exhibits a shape similar to Aegirine due to the presence of only sixfold coordinate Fe^3+^. At the highest pressure, 34.6 GPa in this work, the overall XANES shape of BaFe_4_O_7_ becomes more like Fayalite, significantly different from the marked region in Aegirine as shown in Figure 2e, indicating the presence of sixfold coordination Fe^2+^. To probe the local bonding environment of Fe ions, we performed extended X‐ray absorption fine structure (EXAFS) analysis at the Fe *K*‐edge using a wavelet transform (WT),^[^ ^37^, ^38^ ^]^ as shown in Figure 2f. WT‐EXAFS analysis provides insight into the local bonding configuration in the 2D *K*‐ and *R*‐space, an ideal tool for discriminating atomic pair distribution function with substantial overlapping in *R*‐space. Based on the bond length list from the BaFe_4_O_7_ crystal structure in Table S1 (Supporting Information), we can easily assign the peaks to various bond lengths as shown in Figure 2f. Notably, above 24.3 GPa, the peak for Bond Fe_tet._─Fe disappears, while the peak for Bond Fe_oct._─Fe_oct._ persists, indicating Fe atoms at the tetrahedral site become disorder under high pressure, whereas the Fe atoms at the octahedral site remain still in order status. After decompression to ambient pressure, the Fe─O bonds are distinctly categorized into Fe_oct._─O and Fe_tet._─O. The average bond length of Fe_tet._─O is ≈1.4 Å, much smaller than the 2 Å observed at the first measurement pressure 0.5 GPa, while the bond length of Fe_oct._─O is ≈2.1 Å, exceeding the 2 Å at 0.5 GPa. Given that the ionic radius of Fe decreases with oxidation state increasing, this observation strongly supports the conclusion that the valence state of Fe at the octahedral site after decompression is less than +3, while the valence state of Fe at the tetrahedral sites is higher than +3. This suggests that a charge transfer between the Fe at tetrahedral and octahedral sites occurs upon decompression to ambient pressure.

### 2.2 Charge‐Transfer‐Induced Stabilized Ordered–Disordered Structure and Jahn–Teller Distortion

**Figure 3:**
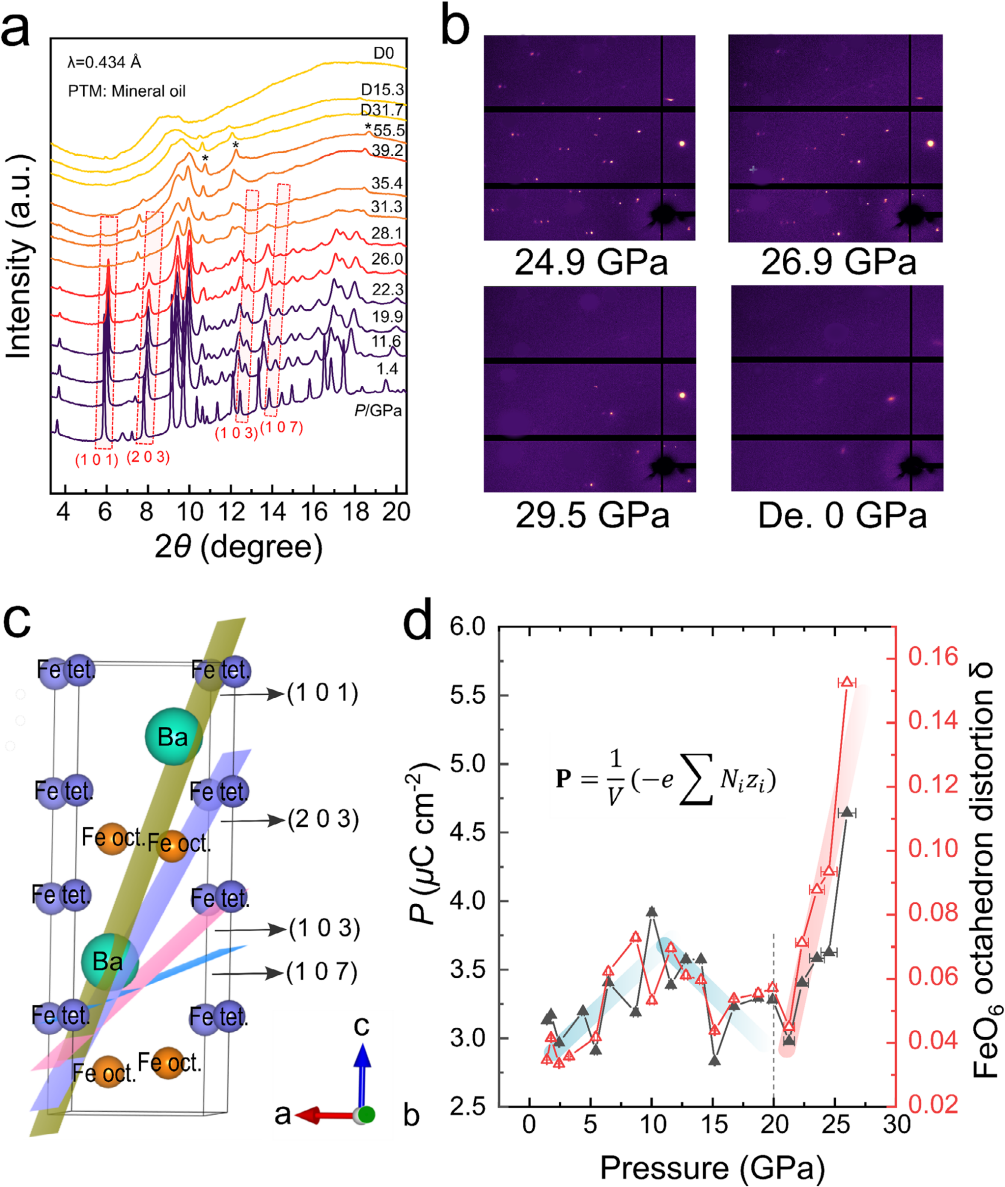
Structural evolution of BaFe_4_O_7_ under high pressure. a) Synchrotron P‐XRD patterns of BaFe_4_O_7_ under various pressures at room temperature. The red dashed box indicates the peaks that disappear with pressure beyond 28.1 GPa. The grey asterisk represents the peak of rhenium. The pressure transmitting medium (PTM) is mineral oil. b) Single crystal X‐ray diffraction patterns under various pressures. c) Structure schematic diagram of the crystal planes (1 0 1) (2 0 3) (1 0 3) and (1 0 7) which disappear under high pressure d) Pressure dependence of *P*_r_ and FeO_6_ octahedron distortion index δ. The pressure error bars are obtained from the pressure gradient shown in Figure S15 (Supporting Information).

At pressure exceeding 20 GPa, the tetrahedral Fe undergoes charge transfer, resulting in oxidation states greater than +3. In certain oxides, higher valence states of Fe beyond Fe^3+^ can be stabilized, particularly when synthesized under highly oxidative conditions.^[^ ^39^, ^40^, ^41^ ^]^ These unusually high oxidation states of Fe are inherently metastable, contributing to long‐range atomic disorder, which helps alleviate electronic instabilities. The structural evolution of BaFe_4_O_7_ upon compression and decompression was investigated using in situ high‐pressure powder and single‐crystal X‐ray diffraction (XRD) measurements (**Figure** **3**a,b). Since this work involves the measurement of ferroelectric properties, a high‐insulation pressure‐transmitting medium is required. To ensure consistency across measurements without compromising the experimental results, mineral oil was primarily used as the pressure medium. In addition, the pressure gradient within the sample chamber was calibrated and indicated in the figures as pressure error bars. To further validate the reliability of the results, Neon was also employed as a comparative pressure‐transmitting medium when necessary. Detailed experimental procedures can be found in the Supporting Information. Below 28.1 GPa, all diffraction peaks of BaFe_4_O_7_ can be indexed to the trigonal *P*3_1_c structure, identical to the ambient pressure crystal structure, indicating no significant structural changes. A typical Rietveld refinement result at 1.4 GPa is shown in Figure S1 (Supporting Information). The shifting of Bragg peaks to higher angles with increasing pressure suggests lattice shrinkage. Above 28.1 GPa, several diffraction peaks become quite broad and weak. The most notable feature is the disappearance of (1 0 1) (2 0 3) (1 0 3) and (1 0 7) reflections. Single crystal XRD measurements at pressures above 29.5 GPa also show the disappearance of these diffraction spots (Figure 3b), further confirming partial structural disorder. Upon further compression to 55.5 GPa, the structure becomes more disordered, as evidenced by a broad background with several weak yet sharp diffraction peaks, indicating that the sample remains a mixed structure of ordered and disordered regions under high pressure. To eliminate the possibility that the partial structural disorder observed under high pressure was influenced by the PTM, we conducted the second round of powder XRD experiments using Neon as the PTM. (Figure S2, Supporting Information) The results were consistent with those obtained with mineral oil. After decompression to 0.05 GPa, the XRD profile was quite different from the pristine one, indicating an irreversible structure transition. The unit cell volume and axis c/a ratio of BaFe_4_O_7_ below 25 GPa were determined through powder Rietveld refinement, as shown in Figure S3 (Supporting Information). The unit cell volume as a function of pressure was plotted and fitted by the second Birch–Murnaghan (B–M) Equation of State (EoS).^[^ ^42^, ^43^ ^]^ Three discontinuities are observed at ≈6, ≈12, and ≈20 GPa, which are probably attributed by structure distortion. Three EoS curves are fitted with the data 1.4–5.4, 6.5–11.6, and 12.8–19.9 GPa, respectively. Likewise, the lattice constant ratio c/a shows a minimum at ≈20 GPa. To further visualize the lattice structure related to the disappeared diffraction peaks after 28.1 GPa, the corresponding crystal planes are marked on the unit cell in Figure 3c. It is evident that the crystal planes associated with the disappearing peaks are all contributed by Fe atoms in the tetrahedral sites, which refers that the FeO_4_ tetrahedra become disordered after 28.1 GPa. As a result of pressure‐induced charge transfer, Fe ions at the octahedral sites shift from +3 oxidation state to +3−Δ state. This change, coupled with partial degeneracy and occupancy of the *d*‐orbital energy levels, derives a spontaneous Jahn–Teller distortion in the FeO_6_ octahedra, which lowers the system's total energy. The increased distortion of the octahedra plays a crucial role in enhancing of ferroelectric polarization of BaFe_4_O_7_. To study the impact of octahedral distortion on ferroelectricity in BaFe_4_O_7_, *P*3_1_ *c* space group is carried out Rietveld refinements for the XRD data to obtain all bond lengths in the FeO_6_ octahedron below 26 GPa. Here, we define the FeO_6_ octahedron distortion index (δ) to quantitatively describe the degree of polyhedron distortion as introduced by Zhao et al.^[^ ^44^ ^]^ The indices are calculated as follows:

(1) $$\delta =\sqrt{\frac{1}{6}\Sigma_{i=1,6}{\left(d_{Fe-O}^{i}-\langle d_{Fe\;-O}\rangle \right)}^{2}}\;$$

In Equation (1), *d*_Fe‐O_ represents the Fe─O bond length within the octahedron. The distortion indices δ increase as the FeO_6_ octahedron undergo more distortion. In ferroelectric crystals, the separation of positive and negative charge centers leads to spontaneous polarization. Using the point charge model,^[^ ^45^ ^]^ we calculated the total polarization *P* (the sum of ionic and electronic contributions) as $P=\frac{1}{V}\;\left(-e\sum N_{i}z_{i}\right)$, *V* is the unit cell volume, *e* is the electron charge, *N*_i_ is the ionic valence state, *z*_i_ is the projection of the atomic position vector in a unit cell along the ***z*** direction. In Figure 3d, the FeO_6_ octahedron distortion δ is plotted along with the polarization *P* as a function of pressure. Below 20 GPa, *δ* first increases (0–12 GPa) and then decreases (12–20 GPa), followed by an abrupt increase between 20 and 25 GPa. Notably, *P* and *δ* follow almost identical trends, suggesting that the enhancement of octahedral distortion plays a significant role in strengthening the ferroelectric polarization of BaFe_4_O_7_.

### 2.3 Bandgap Engineering and Semiconductor Behavior Improvement under Pressure

**Figure 4:**
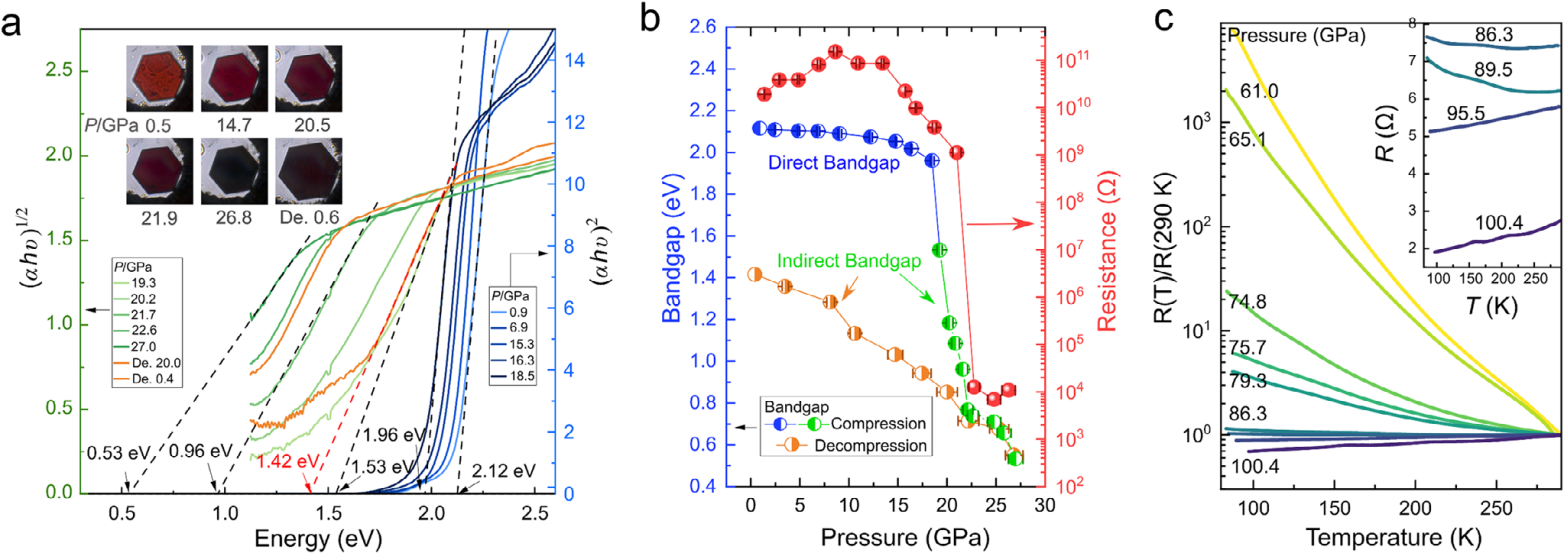
Bandgap engineering and conductivity improvement of BaFe_4_O_7_ under pressure. a) The Tauc plots of UV–vis–NIR absorption spectra at selected pressures. The low pressure (0–18.5 GPa) and high pressure (19.3–27 GPa) were fitted with the direct and indirect bandgap method, respectively. The insets are optical images of the sample with consistent color following the bandgap change. b) Pressure dependence of Resistance along with the bandgap change. During the decompression, an optimized bandgap value 1.42 eV was recovered at ambient pressure. The pressure error bars are obtained from the pressure gradient shown in Figure S15 (Supporting Information). c) Resistance versus temperature curves at selected pressures.

The projected density of states at ambient conditions (Figure S4, Supporting Information) shows that the valence band (VB) consists of O *2p* orbitals while the conduction band (CB) is predominantly contributed by Fe *3d* orbitals, resulting in an optical bandgap (*E*_g_) of 2.2 eV. Previous studies have shown that the splitting of *3d* orbitals in transition metal ions can significantly influence bandgaps. The Fe^3+^ cation has the 3*d* ^5^ valence electronic configuration, and the *d* orbitals of Fe^3+^ evolve into triple‐degenerate *t*_2g_ orbitals and double‐degenerate *e*_g_ orbitals in an octahedral crystal field. At ambient conditions, five electrons of Fe^3+^ occupy all five *3d* orbitals with a high spin state *S *= 5/2 based on the Hund's rule and thereby result in the half‐filled structure. The Fe^2+^ in the sample at high pressure with one more electron occupied *t*_2g_ spin band with *S *= 2 appears above the VB and simultaneously hybridizes with the O *2p* orbit. The maximum VB raises, which is responsible for decreased *E*_g_. Thus, the pressure‐induced intermetallic charge transfer directly will lead to the optimization of optical and electrical properties. The UV–vis–NIR absorption spectroscopy and electric conductivity measurements were employed to evaluate bandgap evolution and electric transport properties under pressure (**Figure** **4**a–c; Figures S5,S6, Supporting Information). To determine the bandgap of BaFe_4_O_7_ under pressure, the optical absorbance spectrum was analysed using the Tauc plot equation:^[^ ^46^, ^47^ ^]^

(2) $${\left(\alpha h\nu \right)}^{\frac{1}{n}}=\;\;A\left(h\nu -E_{g}\right)$$

where, *h* is Planck's constant, *ν* is the frequency, *α* is the absorption coefficient, *E*_g_ is the bandgap, and *A* is the proportional constant. The value of exponential *n* means that it varies with the nature of the sample transition, and when calculating the direct bandgap, *n* is 1/2, and when calculating the indirect bandgap, *n* is 2 theoretically. The inset of Figure 4a shows optical photos taken at selected pressures. At 0.5 GPa, the crystal is transparent with a pale red color and exhibits a sharp absorption edge corresponding to a bandgap of 2.12 eV (Figure S5, Supporting Information). As the pressure increased to 18.5 GPa, the absorption edge displayed a continuous but gradual red shift, indicating a slight narrowing of the bandgap by ≈0.16 eV. Above 18.5 GPa, the absorption curve changes from a steep to a gradual slope, signaling a transition from direct to indirect bandgap. The common approach is further used to distinguish between direct and indirect band gaps by comparing the linearity of Tauc plots, where *n* = 1/2 for direct transitions and *n* = 2 for indirect transitions. The UV–vis–NIR absorption spectrum at 21.7 GPa was selected as a representative dataset. The spectrum was converted into Tauc plots using both the direct and indirect transition models, as shown in Figure S7 (Supporting Information). Linear fitting was applied to the linear regions of both plots. The coefficient of determination (*R* ^2^) obtained from the indirect transition fit (0.999) was higher than that from the direct transition fit (0.996), suggesting that the band gap at pressures above 21.7 GPa is more likely to be an indirect bandgap.

As shown in Figure 4a,b, the low‐pressure region (0–18.5 GPa) exhibits a direct bandgap absorption with minimal changes, while the high‐pressure region (19.33–27 GPa) shows characteristics of indirect bandgap absorption, with a rapid reduction in the bandgap. Simultaneously, conductivity measurements along the normal direction of the crystal flake reveal nearly constant resistance (≈10^9^ Ω) below ≈20 GPa, followed by a dramatic decrease of five orders of magnitude to ≈10^4^ Ω above 20 GPa. (Figure 4b; Figure S6, Supporting Information). The large reduction in the absorption bandgap is favorable for enhancing ferroelectric photovoltaic performance. The crystal color also changed to dark brown, revealing the piezochromism phenomenon. The bandgap undergoes a sharp drop, from 1.96 eV at 18.5 GPa to 0.53 eV at 27 GPa, followed by a partial recovery to 1.42 eV upon pressure release to ambient. The bandgap of 1.42 eV is close to the Shockly–Queissen limit, the ideal bandgap for harvesting the most solar light spectrum. It is notably smaller than the bandgaps of other ferroelectric oxides with small bandgaps under ambient pressure, such as Bi_2_FeCrO_6_ (2 eV),^[^ ^48^ ^]^ Bi(Fe_1‐x_Mn_x_)O_3_ (2.2 eV),^[^ ^49^ ^]^ Ho‐doped Bi_5_Ti_3_FeO_15_ (2.47 eV)^[^ ^50^ ^]^ and KBiFe_2_O_5_ (1.6 eV).^[^ ^51^ ^]^ Electrical conductivity, which significantly impacts the mobility of photogenerated carriers, is a key factor in FPV performance. The resistance versus temperature R(T) curves under pressure up to 100.4 GPa are shown as Figure 4c. BaFe_4_O_7_ maintains semiconducting behavior up to 89.5 GPa due to the presence of disordered regions, which is a necessary condition for applications in the photovoltaic field.

### 2.4 Pressure‐Induced Enhancements of Ferroelectric Polarization and Photocurrent

**Figure 5:**
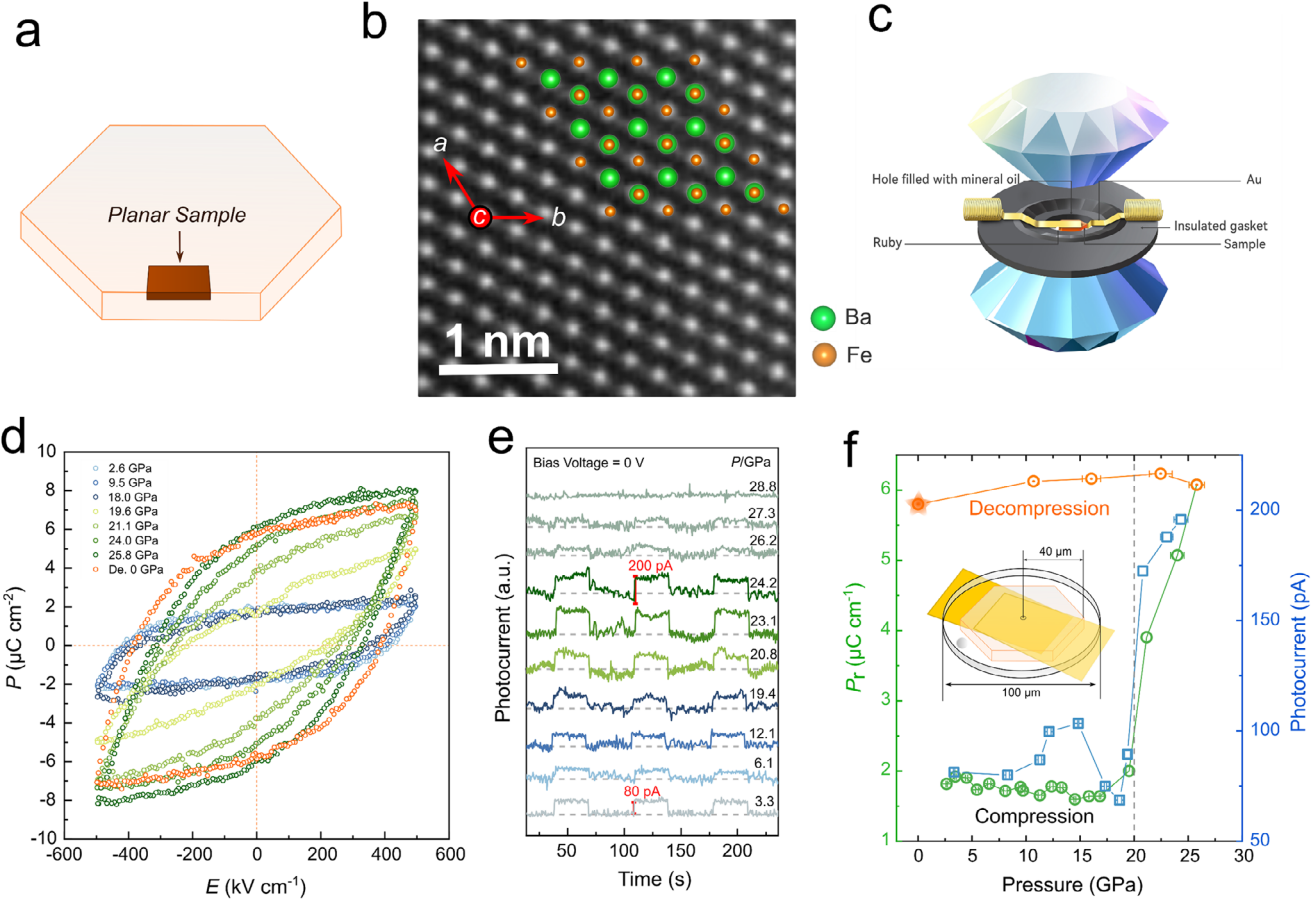
Crystal structure at ambient conditions, ferroelectric photovoltaic effect, and ferroelectric polarization of BaFe_4_O_7_ under pressure. a) Schematic diagram of the planar sample for crystal orientation. b)The high‐angle annular dark‐field scanning transmission electron microscopy (HAADF‐STEM) image of the planar sample from **Figure** **5**a. c) Schematic diagram of the electrode structure for measuring ferroelectric polarization and photocurrent. d) *P‐*‐*E* hysteresis loops of a ferroelectric capacitor of Ag/BaFe_4_O_7_ (10 µm thickness)/Ag at selected pressures. e) The selected zero bias voltage photocurrent intensity following on‐off light, the dashed line represents the current background with the light off. f) Pressure dependence of remnant polarization (*P*_r_), ferroelectric photocurrent intensity for BaFe_4_O_7_. The inset is a schematic diagram of the sample chamber. The pressure error bars are obtained from the pressure gradient shown in Figure S15 (Supporting Information).

To explore the pressure effect on the FPV performance, we conducted the in situ measurements of ferroelectric polarization and photocurrent on a single crystal BaFe_4_O_7_ under pressures up to 28.8 and 25.8 GPa. High‐angle annular dark‐field scanning transmission electron microscopy (HAADF‐STEM) is performed on the surface of a planar sample on the specimen slice to orient the sample and confirm that the normal direction of the specimen slice corresponds to the *c*‐axis direction (shown in Figure 5a,b). The crystal was placed on the culet surface of a diamond anvil, with two electric leads connected with the top and bottom surfaces to measure the photocurrent passing through the sample thickness. The pressure was applied in a Mao‐type diamond anvil cell (DAC), with mineral oil pressure medium, and the pressure was monitored with the Ruby fluorescence method. Figure 5c shows a schematic diagram of the electrode structure. We performed the polarization‐electric field (*P–E*) hysteresis loop measurements with the same sample configuration to 25.8 GPa. Several typical *P–E* loops during the compression process are plotted in Figure 5d and Figure S8 (Supporting Information). At 2.6 GPa, a clear saturated hysteresis loop is observed, and BaFe_4_O_7_ exhibits a noteworthy ferroelectric behavior evidenced by the saturation polarization (*P*_s_) 1.88 µC cm^−2^, remnant polarization (*P*_r_) 1.82 µC cm^−2^ and coercive field (*E*_c_) 418.8 kV cm. As pressure increases, the *P–E* loops undergo systematic changes, with a rapid increase in *P*_r_ occurring ≈20 GPa. Surprisingly, upon decompression, the maximum *P*_r_ reached at 25.8 GPa is largely retained at ambient pressure. The polarization in Figure 3d is calculated from atomic positions using a point‐charge model, while Figure 5f shows experimentally measured *P*_r_. The inflection at ≈12 GPa appears only in the calculated data, likely due to a subtle structural distortion that does not significantly affect the experimental polarization. Factors such as magnetoelectric coupling may also contribute to this discrepancy. Nonetheless, both results consistently show a clear increase in polarization above 20 GPa, confirming the dominant role of structural distortion. To investigate the reason why the *P*_r_ remains at a level comparable to the high polarization under high pressure (above 25 GPa) after decompression to ambient pressure (Figure 5d), The P‐XRD patterns of BaFe_4_O_7_ after pressurizing up to 25.6 GPa and then releasing it to ambient pressure are measured (Figure S9, Supporting Information). At the highest pressure 25.6 GPa, during the compression, the ordered structure is still preserved, although some structural disorder occurs. We previously confirmed that the ordered component consists of Fe atoms at octahedral sites, while the Fe atoms at tetrahedral sites become disordered under high pressure. During the decompression process, the ordered octahedral iron atoms maintain their high‐pressure ordered state, while the tetrahedral iron atoms become more disordered. Since the polarization of BaFe_4_O_7_ is mainly driven by the distortion of the FeO_6_ octahedra, the sample retains a relatively high ferroelectric polarization even after pressure is released.

Figure 5e shows the photocurrent results under a zero‐bias voltage. The photocurrent is ≈80 pA at 3.3 GPa. Remarkably, above 20.8 GPa, the photocurrent has much enhanced by 2.5‐fold, reaching ≈200 pA (*J*_s_ ≈ 8 µA cm^−2^). The photocurrent decreases significantly with pressure beyond 24.2 GPa and disappears completely at 28.8 GPa. The photocurrent suppression is primarily attributed to increased structural disorder, particularly in the FeO_4_ tetrahedral units, as revealed by pressure‐dependent EXAFS data. Above 28.8 GPa, this disorder becomes critical, strongly scattering carriers and disrupting long‐range charge transport, leading to reduced photocurrent. This is further supported by the resistivity increase beyond 28.8 GPa and the lack of significant bandgap trend changes in optical absorption measurements. Thus, the degradation in photovoltaic performance at high pressures is mainly due to disorder‐induced hindrance of carrier transport rather than bandgap variation. This abnormal enhancement raises questions about how it relates to changes in the ferroelectric properties under pressure, we plot the pressure dependence of both *P*_r_ and photocurrent (*I*_pc_) in Figure 5f, where the consistent trends between *P*_r_ and *I*_pc_ are clearly visible. Below ≈20 GPa, both *P*_r_ and *I*_pc_ remain almost constant at low levels. Above ≈20 GPa, *P*_r_ exhibits a rapid threefold increase at 25.8 GPa and maintains this high level throughout the pressure release process to ambient conditions. This enhanced polarization effectively separates electron–hole pairs and induces an enhanced photovoltaic effect.^[^ ^52^ ^]^ This is directly evidenced by the ferroelectric photocurrent boosted to 200 pA at 24.2 GPa, a 2.5‐folds gain from 80 pA at 19.4 GPa. To evaluate the practical photovoltaic performance of BaFe_4_O_7_ under high pressure, the incident photon‐to‐current efficiency (IPCE) is calculated as shown in Figure S10 (Supporting Information). Although the absolute IPCE values remain modest (0.04–0.11%) due to the inherently low conductivity of bulk ferroelectric materials—a significant enhancement is observed with increasing pressure. Notably, when the applied pressure exceeds 20 GPa, the IPCE increases by ≈2.5‐fold compared to the value at ambient conditions. The simultaneous enhancement of remanent polarization and carrier transport under high pressure jointly boosts the FPV performance of BaFe_4_O_7_. Specifically, pressure triggers Fe charge transfer and Jahn–Teller distortion of FeO_6_ octahedra, enhancing resistivity (The resistivity drops by five orders of magnitude at high pressure) and ferroelectric polarization, respectively. These two effects work together: increased polarization enhances carrier separation, and improved conductivity facilitates their transport, leading to a 2.5‐fold increase in photocurrent. Although polarization and conductivity are usually in competition due to structural and electronic constraints, our results demonstrate that pressure‐induced charge transfer above ≈20 GPa effectively overcomes this trade‐off. In 2017, we also studied the simultaneous enhancement of both photovoltaic and ferroelectric properties in KBiFe_2_O_5_ under pressure‐driven phase transitions.^[^ ^53^ ^]^ Yao et al.^[^ ^19^ ^]^ utilized a piston‐cylinder pressure cell to explore the pressure enhancement of ferroelectric properties of CuInP_2_S_6_. These studies underscore the significant potential of using pressure to tune the ferroelectric and photovoltaic properties of materials, highlighting the need for a deeper understanding of the underlying optimization mechanisms.

The magnetic behavior of the multiferroic BaFe_4_O_7_ under high pressure is essential for a comprehensive understanding of its multifunctional properties. At ambient pressure, the previously reported polymorph of BaFe_4_O_7_ was described as an antiferromagnet with a *T*_N_  945 K.^[^ ^54^ ^]^ To investigate the magnetic behavior of BaFe_4_O_7_ under pressure, Fe *K*‐edge X‐ray magnetic circular dichroism (XMCD) spectra were measured at various pressures up to 25.6 GPa (shown in Figure S11, Supporting Information). From 0.2 to 19.4 GPa, the XMCD signal is strong, reflecting a well‐defined magnetic moment on Fe. As pressure increases further, the XMCD intensity becomes nearly undetectable at 25.6 GPa, indicating a significant suppression or complete loss of magnetic ordering. The observed disappearance of the XMCD signal in the 20–25 GPa region suggests a pressure‐induced collapse of long‐range magnetic ordering, likely associated with structural distortions and charge transfer‐induced spin crossover. This implies that a pronounced magnetoelectric interaction may emerge in the critical pressure range, motivating further experimental and theoretical studies.

### 2.5 Stable Order–Disorder Structure upon Pressure Release

The broadening of the diffraction peaks in the powder XRD patterns after pressure release, coupled with XAS data indicating that the disordering of tetrahedral Fe sites remains irreversible, reveals a previously unreported order–disorder structure in BaFe_4_O_7_ following decompression. In this state, Ba and Fe atoms at octahedral sites maintain their ordered configuration, while tetrahedral Fe sites remain disordered, suggesting that a portion of the high‐pressure‐induced structure is retained. This observation underscores a unique structural response to pressure cycling and points to the stability of the order–disorder state under ambient conditions.

**Figure 6:**
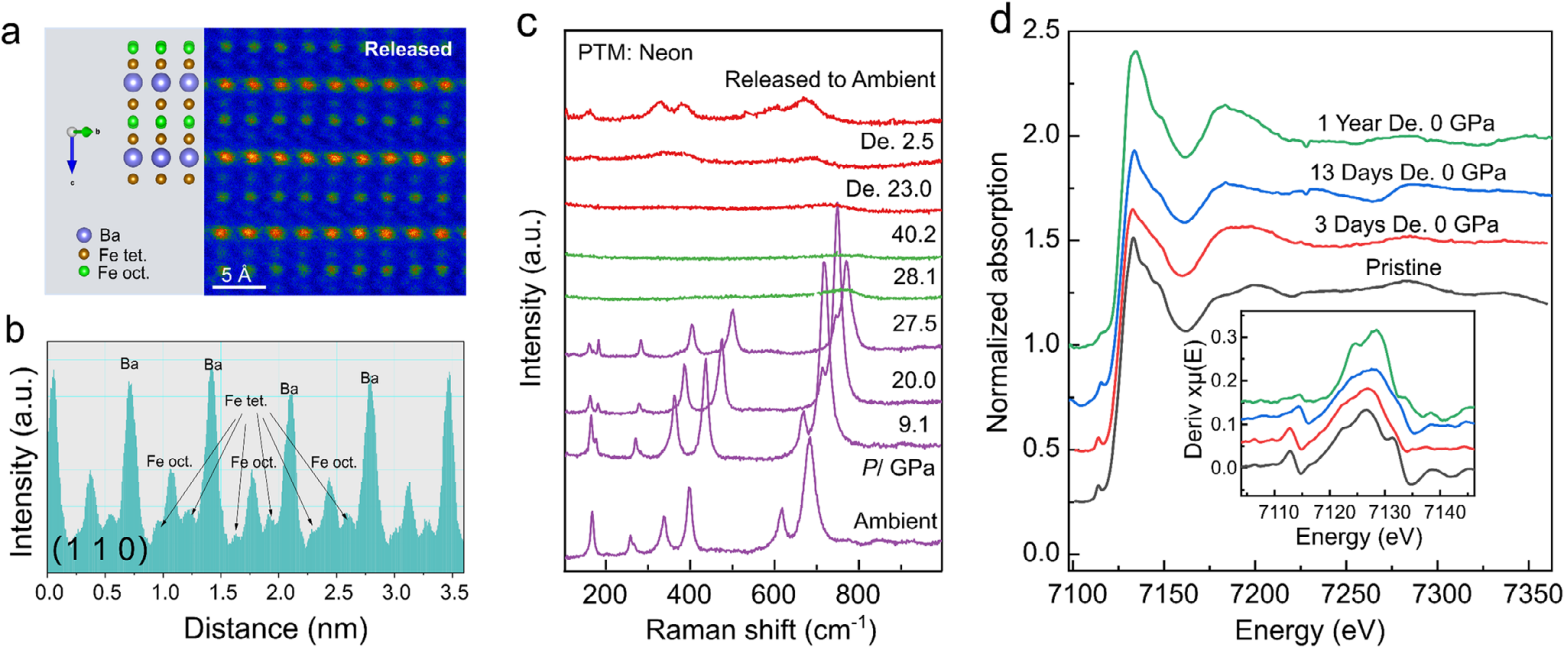
a) The high‐angle annular dark‐field scanning transmission electron microscopy (HAADF‐STEM) image of a pressure‐quenched sample from 40 GPa, with neon as PTM, taken three days after pressure release, in the (1 −1 0) crystallographic plane. The structure model is the ordered structure based on the ambient configuration. b) Intensity‐scan profiles taken from the (1 1 0) plane along [0 0 1] vector across Ba/Fe atomic columns in the pressure‐quenched sample. c) Raman spectra of BaFe_4_O_7_ at selected pressures at room temperature with neon as PTM. d) Fe *K*‐edge X‐ray absorption spectra (XASs) of the pristine sample at ambient conditions and the pressure‐quenched sample from 40 GPa with different on‐shelf times. The XAS of the pressure‐quenched sample is measured 3 days, 13 days, and 1 year after pressure release. The inset shows the first derivative of the XAS spectra.

To further investigate the nature and stability of the order–disorder structure formed upon decompression, we performed microstructural analysis using HAADF‐STEM. The sample, compressed to 40 GPa using neon as the pressure‐transmitting medium (PTM), was subsequently decompressed. Three days after pressure release, HAADF‐STEM imaging was conducted on the recovered sample in the (1 −1 0) crystallographic plane outside the DAC (**Figure** **6**a). The HAADF‐STEM image, along with intensity line profiles taken from the (1 1 0) plane along [0 0 1] vector with Ba/Fe atom columns (Figure 6b), while Ba and Fe atoms at octahedral sites return to an ordered arrangement, the tetrahedral Fe atoms deviate from their original positions and do not revert to the initial structure. These observations provide compelling evidence that the pressure‐induced structural change is irreversible, and that the order–disorder configuration persists under ambient conditions.

In addition to the HAADF‐STEM analysis, in situ Raman spectroscopy was performed during both compression and decompression using neon as the PTM. Raman spectroscopy, highly sensitive to local atomic order, showed that all ambient‐pressure vibrational modes disappeared abruptly between 27.5 and 28.1 GPa, indicating a collapse of long‐range crystallographic order at high pressure (Figure 6c). Notably, this pressure range corresponds to the onset of partial disordering observed in our XRD experiments with mineral oil as the PTM, confirming the reliability of mineral oil in tracking structural transitions. Upon decompression, several Raman modes partially reappeared but did not fully recover, further supporting the irreversible nature of the pressure‐induced structural transition and the stability of the recovered order–disorder structure at ambient conditions. The Raman spectra after releasing from 36–40 GPa showed no significant changes over nine days/2 years and remained consistent with those obtained immediately after pressure release, indicating the long‐time stability of the released structure at ambient conditions (shown in Figure S12, Supporting Information).

The Fe *K*‐edge XAS was collected on 3 days, 13 days, and 1 year after pressure quenching at 40 GPa and compared to that of the pristine sample (Figure 6d). The quenched sample exhibits a notably smoother XAS profile and first‐derivative curve, with a significant reduction in fine structure features. These observations suggest a substantial reduction in local structural order around Fe atoms following high‐pressure quenching and subsequent decompression to ambient conditions. Importantly, this pressure‐induced order–disorder transition remains long‐time stable under ambient conditions, indicating that the structural transformation is thermodynamically favored.

## 3 Conclusion

**Figure 7:**
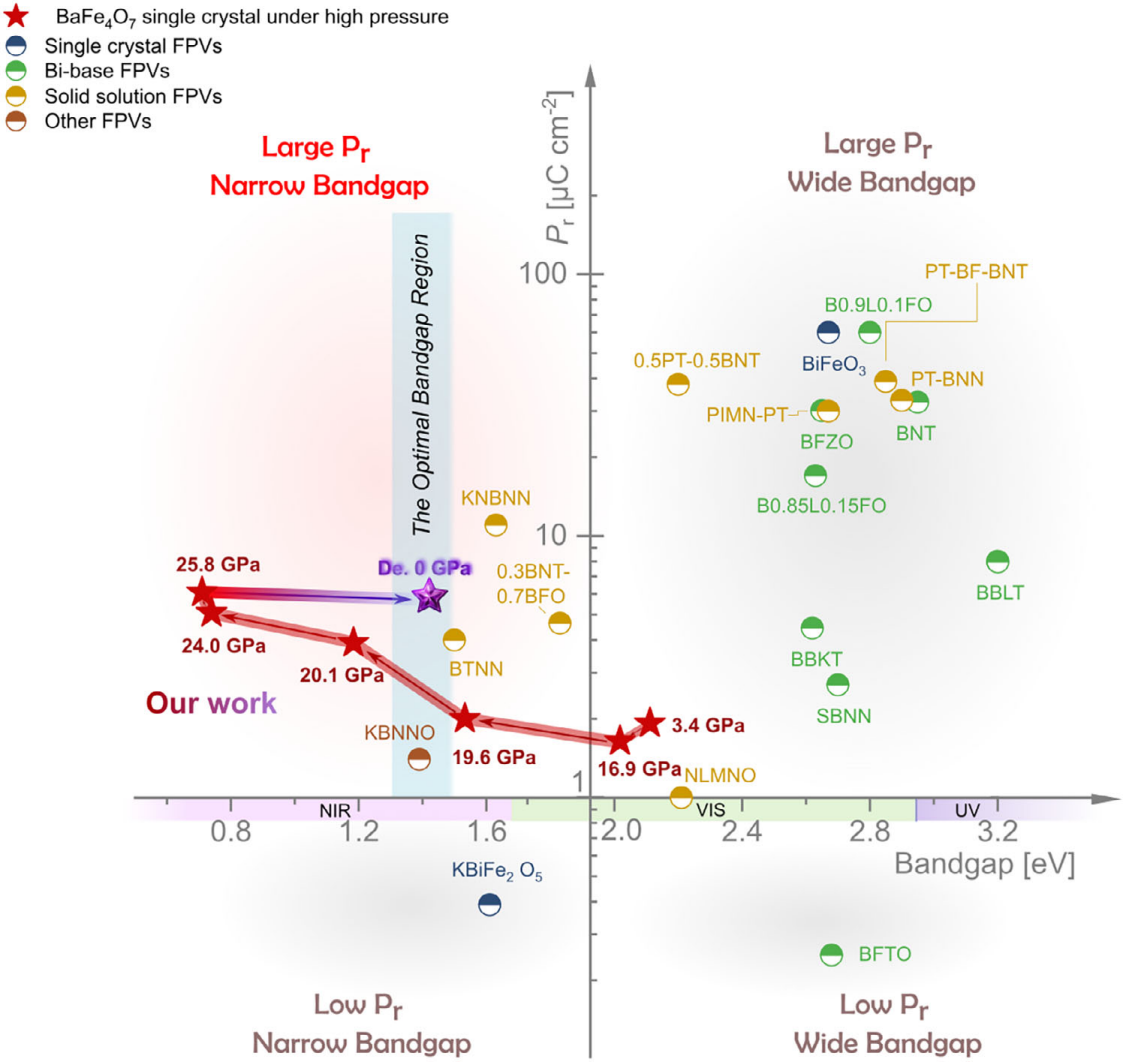
Summary of experimentally reported bandgaps and remanent polarization of FPV. Among the four quarters in bandgap‐*P*_r_ space, our work on BaFe_4_O_7_ demonstrates a unique location with the optimal bandgap for solar light absorption and a large ferroelectric *P*_r_‐value, which gives great potential for practical applications. References are summarized in Table S2 (Supporting Information).

In this work, we present a comprehensive study of the pressure‐driven behavior of BaFe_4_O_7_ revealing a unique interaction between electronic structure, local coordination environment, and ferroelectric–photovoltaic properties. Using high‐resolution Fe *K*‐edge XANES and EXAFS analyses, we observe a pressure‐induced charge transfer beginning ≈20 GPa, which leads to mixed Fe valence states and disordering of tetrahedral FeO_4_ units. These changes are accompanied by a spontaneous Jahn–Teller distortion of the FeO_6_ octahedra between 20 and 25 GPa, resulting in a pronounced enhancement of ferroelectric polarization. High‐pressure bandgap engineering reduces the optical bandgap from 2.12 to 0.53 eV, with a partially recovered value of 1.42 eV upon decompression — approaching the Shockley–Queisser limit and significantly outperforming other known ferroelectric oxides. This modulation of the electronic structure, combining with a five‐order magnitude decrease in resistivity, results in substantial improvements in semiconductor behavior and photovoltaic performance. Upon pressure release, BaFe_4_O_7_ retains its optimized bandgap and maximum polarization *P*_r_, preserving its peak performance, in its ambient state. Notably, the material undergoes a pressure‐induced irreversible order–disorder transition during decompression, when Fe atoms at tetrahedral sites remain disordered, while Fe atoms at octahedral sites retain their long‐range ordered structure. This metastable mixed state persists at ambient conditions. The simultaneous enhancement of light absorption and ferroelectric performance under pressure leads to a 2.5‐fold increase in ferroelectric photocurrent. Compared to thin film FPV materials, the bulk FPV boasts superior mechanical strength, unparalleled chemical stability, reduced production costs, and the ability to bypass substrate growth, positioning them as a powerhouse with immense potential for groundbreaking advancements. In conclusion, we compared the bandgap and polarization constants of these bulk FPVs that have been studied with this work, as shown in **Figure** **7** and Table S2 (Supporting Information). It can be observed that most of the FPVs even those recognized as having application potential, suffer from the issue of incompatible narrow bandgaps and high ferroelectricity. In the four quarters of bandgap‐Polarization *P*_r_ space, the BaFe_4_O_7_ system occupies the unique location with the optimized bandgap for solar absorption and enhanced ferroelectric performance, which provides an effective route for achieving better ferroelectric photovoltaic performance. While this work focuses on hydrostatic pressure as a tuning parameter, similar modulation of electronic structure and ferroelectric properties can also be realized through other external stimuli, including epitaxial strain, chemical substitution (chemical pressure), and uniaxial stress. For example, epitaxial strain has been shown to significantly increase the ferroelectric transition temperature and enhance dielectric properties in SrTiO_3_.^[^ ^55^ ^]^ Similarly, the combination of chemical doping and epitaxial stress improves ferroelectric performance in La‐doped HfO_2_ films.^[^ ^56^ ^]^ Additionally, uniaxial pressure can control domain structures and tune dielectric and piezoelectric responses in ErMnO_3_.^[^ ^57^ ^]^ Chemical pressure effects have also been demonstrated in TbMnO_3_, where magnetic field‐assisted chemical substitution enhances ferroelectric polarization under ambient pressure.^[^ ^58^ ^]^ These findings indicate that pressure‐like effects can be achieved via multiple alternative routes, broadening the applicability of the current results.

## 4 Experimental Section

### Sample Preparation and Characterizations

BaFe_4_O_7_ single crystals were synthesized using a straightforward hydrothermal method, as reported previously.^[^ ^29^ ^]^ To begin, 10 mL of Ba(NO_3_)_2_ (0.2 mol L^−1^) and 40 mL of Fe(NO_3_)_3_ (0.2 mol L^−1^) were mixed in a beaker, followed by continuous stirring with a magnetic stirrer. Next, 60 g of KOH was added to the beaker without interrupting the stirring process. After cooling the reaction mixture to room temperature in air, it was transferred to a 100 mL Teflon‐lined stainless‐steel autoclave, filling it to 80% of its capacity. The autoclave was then heat‐treated at 240 °C for 72 h. After cooling to room temperature, the samples were collected, washed with deionized water, and desiccated at 75 °C in air. Hexagonal single crystals, ranging in size from 20 to 100 µm and exhibiting a reddish‐brown color were obtained, as shown in Figure S13 (Supporting Information). Figure S14 (Supporting Information) displays the X‐ray diffraction (XRD) pattern of the synthesized BaFe_4_O_7_ sample at ambient pressure, which is in excellent agreement with the pattern reported in ref. [29]. At ambient pressure, the XRD of the prepared BaFe_4_O_7_ powder sample was measured using a Malvern Panalytical X‐ray diffractometer with Cu K𝛼 radiation (wavelength 1.5406 Å), operated at 40 kV and 40 mA.

### In Situ High‐Pressure Characterizations—Pressure‐transmitting Medium (PTM) Selection

In this study, ferroelectric polarization measurements were carried out under high pressure, requiring the application of electric fields up to 500 kV cm^−1^ between the sample electrodes. To ensure safe and stable operation under such high electric fields, the pressure‐transmitting medium (PTM) must not only provide good hydrostatic properties but also excellent insulation. Mineral oil was chosen due to its favorable balance of properties: it offers better hydrostatic conditions than silicone oil and exhibits comparable dielectric strength. While gas‐based PTMs provide superior hydrostaticity, their dielectric strength is approximately an order of magnitude lower than that of mineral oil, making them unsuitable for high‐field ferroelectric measurements as shown in Table S3 (Supporting Information). This choice ensured reliable pressure transmission while maintaining electrical insulation integrity throughout the experiments.

To maintain consistency across all high pressure characterization experiments, mineral oil was used as the PTM in all ferroelectric (Figure 5d,f), photocurrent (Figure 5e,f), resistance (Figure 4b,c), UV–vis–NIR absorption spectra (Figure 4a,c) and X‐ray diffraction measurements (Figure 3a,b,d). To demonstrate that mineral oil did not affect the structural changes during pressurization and depressurization, powder XRD (Figure S2, Supporting Information) and single‐crystal Raman spectroscopy (Figure 6c) were conducted using neon as the PTM for comparison.

In the high‐pressure experiments, the pressure within the sample chamber was transmitted from both the center of the diamond culet and the edge of the gasket. A smaller sample chamber led to a more uniform pressure gradient distribution. For these measurements, diamond anvils were consistently used with a 300 µm culet diameter and employed mineral oil as the PTM. The sample chamber, with a diameter of 100 µm, was laser‐drilled into a gasket that was pre‐indented to a thickness of ≈35 µm.

To demonstrate that the pressure gradient within the carefully designed sample chamber did not affect the experimental conclusions in the pressure range (<30 GPa), a pressure standard deviation test was conducted using a 100 µm‐diameter sample chamber in a pre‐indented gasket (35 µm thickness) and pressurized up to 30 GPa. In this test, two tiny ruby spheres were placed—one at the center of the sample chamber and the other 40 µm away from the center. The pressure standard deviation (SD) between the two points was calculated to evaluate the pressure uniformity.^[^ ^59^ ^]^ The standard deviation formula is given by:

(3) $$SD=\sqrt{\frac{1}{N}\sum_{i=1}^{N}{\left(P_{i}-\bar{P}\right)}^{2}}$$

where $\bar{P}$ is the average pressure $\bar{P}=\frac{1}{N}\;\sum_{i\;=1}^{N}P_{i}$. The results are shown in Figure S15 (Supporting Information). At 30 GPa, the SD of the pressure distribution within the sample chamber is only ≈0.8 GPa, and the maximum pressure difference is ≈1.5 GPa, which was well within the acceptable range and did not affect the experimental results.

### High‐Pressure X‐Ray Absorption Spectroscopy (XAS)

The Fe *K*‐edge XAS (Energy = 7112 eV) was performed at the ODE beamline at synchrotron SOLEIL. The ODE beamline was dedicated to energy‐dispersive high‐pressure extended X‐ray absorption fine structure (XAFS) experiments, covering an energy range between 3.5 to 25 keV. The XAFS data were collected with a typical energy step of 0.22 eV per pixel, following calibration of a standard EXAFS spectrum of Fe foil. The beam spot size at ODE was ≈25 µm × 35 µm in FWHM with a tail extending to 70 µm (at 7 keV).

Fine powders of BaFe_4_O_7_ were freshly ground from single crystals and compressed into a pre‐indented Re sample chamber without PTM. Nano‐polycrystalline diamond anvils (NPDs) with a culet diameter of 300 µm and a height of 1.2 mm were used for the high‐pressure experiments at the ODE beamline. The sample chamber has a diameter of ≈70 µm.

An iron foil standard was measured for energy calibration. In situ pressure was controlled using a gas membrane and the PACE5000 (GE) pressure controller. The pressure was calibrated via ruby luminescence.^[^ ^60^ ^]^

### High‐Pressure Synchrotron X‐Ray Diffraction (XRD)

In Figure 3a, in situ high‐pressure angle‐dispersive powder XRD experiments were performed at the 13‐BM‐C station of the Advanced Photon Source (APS), Argonne National Laboratory (ANL) with a wavelength 0.434 Å. The beam size was 15 µm × 15 µm. Fine powders, freshly ground from single crystals, were used for the measurement. Pressure was applied using a pair of diamond anvils with a 300 µm culet size. A Re gasket was pre‐indented to a thickness of ≈40 µm, and a sample chamber with a diameter of 100 µm was drilled at the center of the indentation using a laser. A powder sample, ≈30 µm a diameter was loaded into the chamber. A small ruby sphere was placed adjacent to the sample for pressure calibration,^[^ ^60^ ^]^ and mineral oil was used as the PTM.

In Figure 3b, in situ high‐pressure single crystal XRD experiments were carried out at the 13‐BM‐C station of the Advanced Photon Source (APS), Argonne National Laboratory (ANL) with a wavelength 0.434 Å. The beam size is 15 µm × 15 µm. Pressure was applied using a pair of diamond anvils with a 300 µm culet size. A Re gasket was pre‐indented to a thickness of ≈40 µm. A sample chamber with a diameter of 100 µm was drilled at the center of the indentation using a laser. A single crystal sample with a diameter of ≈20 µm was loaded into the chamber. A small ruby sphere was placed adjacent to the sample for pressure calibration,^[^ ^59^ ^]^ and mineral oil was used as the PTM.

In Figure S2 (Supporting Information), the in situ high‐pressure angle‐dispersive powder XRD experiments were carried out at the P02.2 beamline of Deutsches Elektronen‐Synchrotron (DESY) with a wavelength 0.2904 Å. The beam size is 2 µm × 2 µm. Pressure was applied using a pair of diamond anvils with a 150 µm culet size. A Re gasket was pre‐indented to a thickness of ≈30 µm. A sample chamber with a diameter of 60 µm was drilled at the center of the indentation using a laser. A powder sample with a diameter of ≈30 µm was loaded into the chamber. A small Au sheet was placed adjacent to the sample for pressure calibration, and neon was used as the PTM.

In Figure S9 (Supporting Information), the in situ high‐pressure angle‐dispersive powder XRD experiments were carried out at the BL17UM station of the Shanghai Synchrotron Radiation Facility (SSRF) with a wavelength of 0.4838 Å. The beam size is 10 µm × 6 µm. Pressure was applied using a pair of diamond anvils with a 300 µm culet size. A Re gasket was pre‐indented to a thickness of ≈40 µm. A sample chamber with a diameter of 100 µm was drilled at the center of the indentation using a laser. A powder sample with a diameter of ≈30 µm was loaded into the chamber. A small ruby sphere was placed adjacent to the sample for pressure calibration,^[^ ^60^ ^]^ and mineral oil was used as the PTM.

Rietveld refinements were conducted with the general structure analysis system and graphical user interface EXPGUI package.^[^ ^61^ ^]^ The ambient pressure crystalline structural parameters were derived from the research of Zhang et al.^[^ ^29^ ^]^

### High‐Pressure UV–Vis–NIR Absorption Spectroscopy

UV–vis–NIR absorption spectra were collected using a custom‐built microprobe spectroscopy system (Gora‐UVN‐FL, manufactured by Ideaoptics, Shanghai, China) which was equipped with a xenon light source with a wavelength range of 200 to 1700 nm. Pressure was applied using a diamond anvil cell with a 300 µm culet diamond. A Re gasket was pre‐indented to a thickness of ≈35 µm, and a sample chamber with a diameter of 100 µm was drilled at the center of the indentation using a laser. A single crystal sample with a diameter of ≈60 µm was loaded into the chamber. A small ruby sphere was placed adjacent to the sample for pressure calibration,^[^ ^60^ ^]^ and mineral oil was used as the PTM.

### High‐Pressure Resistance Measurements

In Figure 4b, the resistance measurements of the BaFe_4_O_7_ single crystal were performed in DAC with culets of 300 µm. An 80 µm diameter BaFe_4_O_7_ single crystal was loaded into the sample chamber ≈100 µm in the stainless steel gasket insulated with cubic boron nitride (cBN), and contacted two gold wire electrodes on each top and bottom side of sample along the *c*‐axis. The mineral oil was used as a PTM. The impedance meter (Model CS353, Wuhan CorrTest Instr. Corp., Ltd.; or Model Zahner, Zahner) was used to conduct the EIS measurements in a frequency range from 0.01 Hz to 1 MHz (signal amplitude 20 mV). The EIS data were fitted using an equivalent circuit with the help of the Zview software.^[^ ^62^, ^63^ ^]^

In Figure 4c, the low‐temperature resistance measurements of the BaFe_4_O_7_ single crystal were performed in DAC with culets of 150 µm. Load a 40 µm diameter BaFe_4_O_7_ single crystal into the sample chamber ≈60 µm in the Re gasket insulated with cubic boron nitride (cBN) and contact two gold wire electrodes on each top and bottom side of the sample along the *c*‐axis. The mineral oil was used as a PTM. The resistances between the two electrodes were determined using a Keithley 2182A nanovoltmeter; a Keithley 6221 DC/AC source was used to provide the direct current for the resistance measurements. The sample was initially cooled using liquid nitrogen. Once the temperature reached ≈90 K, resistance measurements were conducted during the subsequent warming process back to room temperature. Pressure calibration was performed using the Raman second‐order peak located ≈1334 cm^−1^ under ambient pressure with a diamond.

### High‐Pressure Ferroelectric Measurements

The *P*–*E* hysteresis loop of BaFe_4_O_7_ single crystal was measured by a home‐made system based on the Sawyer–Tower circuit^[^ ^63^ ^]^ with varying the amplitude of the applied voltage at 1 kHz. The circuit diagram for measuring the *P–E* hysteresis loop is shown in Figure S16 (Supporting Information). Pressure was applied using a diamond anvil with a 300 µm culet. A Re gasket, electrically insulated with cBN, was pre‐indented to a thickness of 35 µm. A sample chamber with a diameter of 100 µm was drilled at the center of the indentation using a laser. A single‐crystal sample with a diameter of 80 µm was loaded into the chamber, with two gold wire electrodes with a width of 60 µm attached to its top and bottom surfaces using silver paste. A small ruby sphere was placed adjacent to the sample for pressure calibration,^[^ ^60^ ^]^ and mineral oil was used as the PTM.

### High‐Pressure Photocurrent

Pressure was applied using a diamond anvil with a 300 µm culet. A Re gasket, electrically insulated with cBN), was pre‐indented to a thickness of 35 µm. A sample chamber with a diameter of 100 µm was drilled at the center of the indentation using a laser. A single‐crystal sample with a diameter of 80 µm was loaded into the chamber, with two gold wire electrodes attached to its top and bottom surfaces using silver paste. A small ruby sphere was placed adjacent to the sample for pressure calibration,^[^ ^59^ ^]^ and mineral oil was used as the PTM. The light source used for the measurement was a 10 W LED lamp with a wavelength range of 400 to 410 nm. A high resistance meter (Keithley 6517B, A Tektronix Co., Ltd.) was used to monitor the photocurrent under zero‐bias voltage. The sample was repeatedly illuminated by 30 s pulsed light every 40 s.

The *P*–*E* hysteresis loop and ferroelectric photocurrent data were collected in two separate high‐pressure experiments. Due to setup limitations, they could not be measured simultaneously, which led to slight differences in the selected pressure points. However, both experiments followed consistent procedures, and the trends were comparable.

### High‐Pressure Raman Spectroscopy

High‐pressure Raman spectroscopy under high pressure was measured by a Raman spectrometer belonging to HPSTAR using a 532 nm excitation laser. The laser size is 2 µm × 2 µm. Pressure was applied using a diamond anvil with a 300 µm culet. A Re gasket was pre‐indented to a thickness of ≈40 µm. A sample chamber with a diameter of 100 µm was drilled at the center of the indentation using a laser. A single crystal sample with a diameter of ≈30 µm was loaded into the chamber. A small Ruby was placed adjacent to the sample for pressure calibration, and neon was used as the PTM.

### IPCE Measurement and Calculation

The incident photon‐to‐current efficiency (IPCE) was evaluated under monochromatic illumination at a fixed wavelength of 405 nm with an incident power density of 56 mW cm^−2^.

The IPCE was calculated using the following formula:

(4) $$\mathrm{IPCE}\;=\frac{1240\times J_{ph}}{\lambda \;\times P_{in}}\times 100\%$$

where *J_ph_* is the measured photocurrent density (in mA cm^−2^), *λ* = 405 nm is the wavelength of the incident light, and *P_in_* = 56 mW cm^−2^ is the incident power density. This approach allowed direct comparison of photoresponse performance under varying pressure conditions.

### Characterization under Ambient Condition—High‐Angle Annual Dark‐Field Scanning Transmission Electron Microscopy (HAADF‐STEM)

HAADF‐STEM of the ambient sample and pressure quenched sample were performed using a 200 kV JEM‐ARM200F (JEOL NEOARM) equipped with a spherical‐aberration corrector (CEOS Gmbh). The convergence angle was chosen of 30 mrad. The annular‐dark field detector inner and outer angles were 68 and 280 mrad, respectively.

### First‐Principles Calculation

The DFT calculations were performed using the Vienna ab initio simulation package) with generalized gradient approximation (GGA) of the Perdew–Burke–Ernzerhof (PBE) exchange‐correlation functional and projector augmented wave potentials.^[^ ^64^, ^65^, ^66^, ^67^, ^68^, ^69^ ^]^ To account for the possible effect of strongly correlated electrons, GGA + U calculations^[^ ^70^, ^71^ ^]^ were performed for band structure and density of states using the Coulomb interaction parameter (4 eV for Fe elements). The cutoff energy was set to 600 eV and a 0.03 Å^−1^ Monkhorst–Pack k‐points grid was used for structural relaxations, in which the criterion for energy convergence was 10^−7^ eV.

## Conflict of Interest

The authors declare no conflict of interest.

## Author Contributions

W.G.Y. and G.H.Z. conceived the idea and experimental design. G.H.Z. was responsible for synthesizing material. J.Y.G. conducted all in situ high‐pressure measurements, and both J.Y.G. and B.H.W. analyzed and interpreted the experimental data. B.H.W., N.N.L., L.M.Y., and X.Q.L. assisted with sample loading and electrodes building in DAC. S.P. supported HAADF‐STEM. N.N.L., M.T.L., W.G.Y., A.N., and Q.Y.K. contributed to the in situ high pressure X‐ray absorption measurements. D.Z.Z., N.N.L., B.H.W., K.Z., W.G.Y., and G.W. assisted with in situ high pressure X‐ray diffraction. D.Z.Z., S. P., and K.J.B. supported crystal structure analysis. K.J.B., Y.H.M., and X.J.L. assisted with the UV–vis–NIR absorbance measurements. X.Z., T.L., and T.Z. assisted with *P*‐*E* hysteresis loop measurement. J.Y.G. and W.G.Y. wrote the manuscript. All authors contributed to the discussion of the results and revision of the manuscript.

## Supporting information



Supporting Information

## Data Availability

The data that support the findings of this study are available from the corresponding author upon reasonable request.

## References

[1] S. Y. Yang , J. Seidel , S. J. Byrnes , P. Shafer , C. H. Yang , M. D. Rossell , P. Yu , Y. H. Chu , J. F. Scott , J. W. Ager , L. W. Martin , R. Ramesh , Nat. Nanotechnol. 2010, 5, 143. 20062051 10.1038/nnano.2009.451

[2] T. Choi , S. Lee , Y. J. Choi , V. Kiryukhin , S. W. Cheong , Science. 2009, 324, 63. 19228998 10.1126/science.1168636

[3] S. W. Cheong , H. T. Yi , T. Choi , A. Hogan , in Frontiers in Electronic Materials: A Collection of Extended Abstracts of the Nature Conference Frontiers in Electronic Materials (eds: J. D. Y. R. M 2012, 10.1002/9783527667703.ch5

[4] S. Wemple , Phys. Rev. B. 1970, 2, 2679.

[5] J. F. Scott , Jpn. J. Appl. Phys. 1999, 38, 2272.

[6] C. X. Li , X. Li , X. Y. Chen , C. Chen , L. Zhao , N. Ma , J. Mater. Chem. C. 2023, 11, 12561.

[7] C. Gao , W. L. Li , L. Jing , Z. Wang , L. Shi , J. Sheng , L. D. Wang , Y. Zhao , W. D. Fei , Adv. Funct. Mater. 2023, 33, 2213178.

[8] J. Wang , H. B. Huang , W. Q. He , Q. H. Zhang , D. N. Yang , Y. L. Zhang , R. R. Liang , C. S. Wang , X. Q. Ma , L. Gu , L. Q. Chen , C. W. Nan , J. X. Zhang , ACS Appl. Mater. Interfaces 2017, 9, 24704. 28686410 10.1021/acsami.7b05138

[9] J. Q. Ding , R. Q. Guo , J. C. Hu , G. Q. Xi , Y. Lu , J. J. Tian , L. X. Zhang , Appl. Surf. Sci. 2022, 606, 154898.

[10] X. X. Cui , Y. Li , X. W. Li , X. H. Hao , J. Mater. Chem. C. 2020, 8, 1359.

[11] X. D. Liu , J. Tu , Y. W. Fang , G. Q. Xi , H. R. Li , R. Wu , X. Q. Liu , D. F. Lu , J. S. He , J. W. Zhang , J. J. Tian , L. X. Zhang , J. Am. Chem. Soc. 2024, 146, 13934. 38741463 10.1021/jacs.4c01702

[12] Z. Fei , W. Zhao , T. A. Palomaki , B. Sun , M. K. Miller , Z. Zhao , J. Yan , X. Xu , D. H. Cobden , Nature 2018, 560, 336. 30038286 10.1038/s41586-018-0336-3

[13] S. Shi , G. Hu , M. Wang , H. Wu , X. Liu , H. Wang , J. Xu , L. Yang , W. Qiu , J. Electron. Mater. 2022, 51, 6913.

[14] B. M. Fregoso , T. Morimoto , J. E. Moore , Phys. Rev. B. 2017, 96, 075421.

[15] A. M. Schankler , L. Gao , A. M. Rappe , J. Phys. Chem. Lett. 2021, 12, 1244. 33497221 10.1021/acs.jpclett.0c03503

[16] M.‐R. Li , U. Adem , S. R. C. McMitchell , Z. Xu , C. I. Thomas , J. E. Warren , D. V. Giap , H. Niu , X. Wan , R. G. Palgrave , F. Schiffmann , F. Cora , B. Slater , T. L. Burnett , M. G. Cain , A. M. Abakumov , G. van Tendeloo , M. F. Thomas , M. J. Rosseinsky , J. B. Claridge , J. Am. Chem. Soc. 2012, 134, 3737. 22280499 10.1021/ja208395z PMC3693400

[17] S. Ju , T.‐Y. Cai , H.‐S. Lu , C.‐D. Gong , J. Am. Chem. Soc. 2012, 134, 13780. 22823905 10.1021/ja305167h

[18] X.‐J. Chen , V. V. Struzhkin , Y. Yu , A. F. Goncharov , C.‐T. Lin , H.‐k. Mao , R. J. Hemley , Nature 2010, 466, 950. 20725035 10.1038/nature09293

[19] X. Yao , Y. Bai , C. Jin , X. Zhang , Q. Zheng , Z. Xu , L. Chen , S. Wang , Y. Liu , J. Wang , J. Zhu , Nat. Commun. 2023, 14, 4301. 37463932 10.1038/s41467-023-40075-6 PMC10354068

[20] M. Azuma , S. Carlsson , J. Rodgers , M. G. Tucker , M. Tsujimoto , S. Ishiwata , S. Isoda , Y. Shimakawa , M. Takano , J. P. Attfield , J. Am. Chem. Soc. 2007, 129, 14433. 17973381 10.1021/ja074880u

[21] Z. Liu , Y. Sakai , J. Yang , W. Li , Y. Liu , X. Ye , S. Qin , J. Chen , S. Agrestini , K. Chen , S.‐C. Liao , S.‐C. Haw , F. Baudelet , H. Ishii , T. Nishikubo , H. Ishizaki , T. Yamamoto , Z. Pan , M. Fukuda , K. Ohashi , K. Matsuno , A. Machida , T. Watanuki , S. I. Kawaguchi , A. M. Arevalo‐Lopez , C. Jin , Z. Hu , J. P. Attfield , M. Azuma , Y. Long , J. Am. Chem. Soc. 2020, 142, 5731. 32083872 10.1021/jacs.9b13508

[22] Y.‐w. Long , T. Kawakami , W.‐t. Chen , T. Saito , T. Watanuki , Y. Nakakura , Q.‐q. Liu , C.‐q. Jin , Y. Shimakawa , Chem. Mater. 2012, 24, 2235.

[23] C. Zhu , J. Yang , P. Shan , M.‐H. Zhao , S. Zhao , C. Pei , B. Zhang , Z. Deng , M. Croft , Y. Qi , L. Yang , Y. Wang , X. Kuang , L. Jiang , D.‐X. Yao , J.‐G. Cheng , M.‐R. Li , CCS Chem 2023, 5, 934.

[24] P. E. R. Blanchard , K. W. Chapman , S. M. Heald , M. Zbiri , M. R. Johnson , B. J. Kennedy , C. D. Ling , Inorg. Chem. 2016, 55, 5649. 27187072 10.1021/acs.inorgchem.6b00718

[25] M. Abramchuk , C. Ozsoy‐Keskinbora , J. W. Krizan , K. R. Metz , D. C. Bell , F. Tafti , J. Am. Chem. Soc. 2017, 139, 15371. 28981260 10.1021/jacs.7b06911

[26] K. Bu , Q. Hu , X. Qi , D. Wang , S. Guo , H. Luo , T. Lin , X. Guo , Q. Zeng , Y. Ding , F. Huang , W. Yang , H.‐K. Mao , X. Lu , Nat. Commun. 2022, 13, 4650. 35945215 10.1038/s41467-022-32419-5 PMC9363411

[27] L. Wang , B. Liu , H. Li , W. Yang , Y. Ding , S. V. Sinogeikin , Y. Meng , Z. Liu , X. C. Zeng , W. L. Mao , Science 2012, 337, 825. 22904007 10.1126/science.1220522

[28] Q. Zeng , H. Sheng , Y. Ding , L. Wang , W. Yang , J.‐Z. Jiang , W. L. Mao , H.‐K. Mao , Science 2011, 332, 1404. 21680837 10.1126/science.1200324

[29] G. Zhang , J. Hou , M. Zhu , G. Huang , D. Li , Y. Fang , T. Zeng , J. Mater. Chem. C 2020, 8, 16234.

[30] T. E. Westre , P. Kennepohl , J. G. DeWitt , B. Hedman , K. O. Hodgson , E. I. Solomon , J. Am. Chem. Soc. 1997, 119, 6297.

[31] W. E. Jackson , J. M. de Leon , G. E. Brown , G. A. Waychunas , S. D. Conradson , J. M. Combes , Science 1993, 262, 229. 17841870 10.1126/science.262.5131.229

[32] E. W. White , H. A. McKinstry , Adv. X‐Ray Anal. 1965, 9, 376.

[33] U. Srivastava , H. J. C. C. R. Nigam , Coord. Chem. Rev. 1973, 9, 275.

[34] J. Wong , F. Lytle , R. Messmer , D. J. P. R. B. Maylotte , Phys. Rev. B 1984, 30, 5596.

[35] G. Calas , J. J. S. s. c. Petiau , Solid State Commun. 1983, 48, 625.

[36] C. Henderson , G. Cressey , S. J. R. P. Redfern , Radiat. Phys. Chem. 1995, 45, 459.

[37] H. Funke , M. Chukalina , A. C. Scheinost , J. Synchrotron Radiat. 2007, 14, 426. 17717385 10.1107/S0909049507031901

[38] H. Funke , A. C. Scheinost , M. Chukalina , Phys. Rev. B 2005,71, 094110.

[39] J. B. MacChesney , R. C. Sherwood , J. F. Potter , J. Chem. Phys. 1965, 43, 1907.

[40] M. Takano , N. Nakanishi , Y. Takeda , S. Naka , T. Takada , Mater. Res. Bull. 1977, 12, 923.

[41] Y. Takeda , S. Naka , M. Takano , T. Shinjo , T. Takada , M. Shimada , Mater. Res. Bull. 1978, 13, 61.

[42] F. D. Murnaghan , Proc. Natl. Acad. Sci. U. S. A. 1944, 30, 382. 16588670 10.1073/pnas.30.12.382 PMC1078732

[43] F. Birch , Phys. Rev. 1947, 71, 809.

[44] Y. Zhao , D. J. Weidner , J. B. Parise , D. E. Cox , Phys. Earth Planet. Inter. 1993, 76, 1.

[45] R. Resta , Rev. Mod. Phys. 1994, 66, 899.

[46] N. F. Mott , Philos. Mag. 1971, 24, 1.

[47] R. A. Huggins , Ionics 2002, 8, 300.

[48] R. Nechache , C. Harnagea , S. Licoccia , E. Traversa , A. Ruediger , A. Pignolet , F. Rosei , Appl. Phys. Lett. 2011, 98, 202902.

[49] X. S. Xu , J. F. Ihlefeld , J. H. Lee , O. K. Ezekoye , E. Vlahos , R. Ramesh , V. Gopalan , X. Q. Pan , D. G. Schlom , J. L. Musfeldt , Appl. Phys. Lett. 2010, 96, 192901.

[50] Y. Bai , J. Chen , X. Wu , S. Zhao , J. Phys. Chem. C. 2016, 120, 24637.

[51] G. Zhang , H. Wu , G. Li , Q. Huang , C. Yang , F. Huang , F. Liao , J. Lin , Sci. Rep. 2013, 3, 1265. 23405279 10.1038/srep01265 PMC3569630

[52] Y. Chen , J. Chen , S. Yang , Y. Li , X. Gao , M. Zeng , Z. Fan , X. Gao , X. Lu , J. Liu , Mater. Res. Bull. 2018, 107, 456.

[53] G. Zhang , F. Liu , T. Gu , Y. Zhao , N. Li , W. Yang , S. Feng , Adv. Electron. Mater. 2017, 3, 1600498.

[54] T. Ferreira , G. Morrison , W. M. Chance , S. Calder , M. D. Smith , Chem. Mater. 2017, 29, 2689.

[55] J. H. Haeni , P. Irvin , W. Chang , R. Ueckeret , P. Reiche , Y. L. Li , S. Choudhury , W. Tian , M. E. Hawley , B. Craigo , A. K. Tagantsev , X. Q. Pan , S. K. Streiffer , L. Q. Chen , S. W. Kirchoefer , J. Levy , D. G. Schlom , Nature 2004, 430, 758. 15306803 10.1038/nature02773

[56] T. Song , H. Tan , A.‐C. Robert , S. Estandia , J. Gázquez , F. Sánchez , I. Fina , Appl. Mater. Today. 2022, 29, 101621.

[57] O. W. Sandvik , A. Merlin Muller , H. W. Ånes , M. Zahn , J. He , M. Fiebig , T. Lottermoser , T. Rojac , D. Meier , J. Schultheiß , Nano Lett. 2023, 23, 6994. 37470766 10.1021/acs.nanolett.3c01638 PMC10416345

[58] N. Terada , D. D. Khalyavin , P. Manuel , T. Osakabe , A. Kikkawa , H. Kitazawa , Phys. Rev. B 2016, 93, 081104.

[59] S. Klotz , J.‐C. Chervin , P. Munsch , L. G. Marchand , J. Phys. D: Appl. Phys. 2009, 42, 075413.

[60] H.‐K. Mao , P. M. Bell , Science 1976, 191, 851. 17730998 10.1126/science.191.4229.851

[61] A. C. Larson , R. B. V. Dreele , B. H. Toby , General structure analysis system ‐ GSAS/EXPGUI[J] 1994.

[62] J. Tauc , R. Grigorovici , A. Vancu , Phys. Status Solidi B 1966, 15, 627.

[63] M. Moździerz , J. Dąbrowa , A. Stępień , M. Zajusz , M. Stygar , W. Zając , M. Danielewski , K. Świerczek , Acta Mater. 2021, 208, 116735.

[64] C. B. Sawyer , C. Tower , Phys. Rev. 1930, 35, 269.

[65] G. Kresse , J. Furthmüller , Comput. Mater. Sci. 1996, 6, 15.

[66] G. Kresse , J. Furthmüller , Phys. Rev. B 1996, 54, 11169. 10.1103/physrevb.54.11169 9984901

[67] G. Kresse , D. Joubert , Phys. Rev. B 1999, 59, 1758.

[68] J. P. Perdew , K. Burke , Y. Wang , Phys. Rev. B 1996, 54, 16533. 10.1103/physrevb.54.16533 9985776

[69] J. P. Perdew , Y. Wang , Phys. Rev. B 1992, 45, 13244. 10.1103/physrevb.45.13244 10001404

[70] V. I. Anisimov , J. Zaanen , O. K. Andersen , Phys. Rev. B 1991, 44, 943. 10.1103/physrevb.44.943 9999600

[71] A. I. Liechtenstein , V. I. Anisimov , J. Zaanen , Phys. Rev. B 1995, 52, R5467. 10.1103/physrevb.52.r5467 9981806

