# Supplementary material for: Order–Disorder Phase Stabilization by Pressure‐Induced Charge Transfer Enhances the Ferroelectric Photovoltaic Effect in Multiferroic BaFe4O7
Source: Adv Sci (Weinh). 2025 Aug 19;12(42):e11022. doi: 10.1002/advs.202511022 (PMC12622448; doi:10.1002/advs.202511022)
Supplement: Supplementary file 1 — Supporting Information [file ADVS-12-e11022-s001.docx]

Supporting Information

Order–Disorder Phase Stabilization by Pressure-Induced Charge Transfer Enhances the Ferroelectric Photovoltaic Effect in Multiferroic BaFe_4_O_7_

Jiayi Guan, Bihan Wang, Nana Li, Shang Peng, Ganghua Zhang,* Limin Yan, Xuqiang Liu, Kai Zhang, Mingtao Li, Adama N-Diaye, Qingyu Kong, Dongzhou Zhang, Xu Zhao, Ting Liu, Kejun Bu, Yuhong Mao, Gui Wang, Xujie Lü, Xiang Li, Tao Zeng* and Wenge Yang*

**Figure S1** Typical Rietveld refinements of BaFe_4_O_7_ at low-pressure phase (1.4 GPa) and the corresponding refined structure.

**Figure S2** Synchrotron P-XRD patterns of BaFe_4_O_7_ with neon as the pressure transmitting medium.

**Figure S3**. Pressure dependence of Unit-cell Volumn below 26.0 GPa, and the dashed line represents the Birch−Murnaghan third-order EoS fit to the Pressure−Volumn data of the compression below 26.0 GPa.

**Figure S4.** Projected density of states (DOS) for BaFe_4_O_7_ at ambient conditions.

**Figure S5**. UV−vis-NIR absorption spectra of BaFe_4_O_7_ at selected pressures at room temperature.

**Figure S6.** EIS data (points) and the corresponding fittings(curves) of BaFe_4_O_7_ under high pressure

**Figure S7.** Tauc plots constructed using direct and indirect transition methods at 21.7 GPa.

**Figure S8.** *P*-*E* hysteresis loops of a ferroelectric capacitor of BaFe_4_O_7_ (10 µm thick) during the decompression process.

**Figure S9.** Synchrotron P-XRD patterns of BaFe_4_O_7_ during compression and decompression process at room temperature.

**Figure S10.** Incident photon-to-current efficiency (IPCE) of BaFe_4_O_7_ under high pressure.

**Figure S11.** Fe *K*-edge X-ray absorption near edge structure (XANES) and X-ray magnetic circular dichroism (XMCD) spectra at selected pressures at room temperature.

**Figure S12** Raman spectra of BaFe_4_O_7_ at selected pressures and time-dependent Raman spectra of the sample released from 40 GPa.

**Figure S13.** Optical microscopy image of BaFe_4_O_7_.

**Figure S14.** The measured XRD pattern at ambient conditions.

**Figure S15**. Pressure dependence of hydrostaticity inside the sample chamber of a diamond anvil cell (DAC) with mineral oil as the pressure-transmitting medium (PTM).

**Figure S16** The circuit diagram for measuring the *P-E* hysteresis loop.

**Table S1.** The Fe-O and Fe-Fe bond length range in ambient BaFe_4_O_7_.

**Table S2.** Literature Data of the bulk PFVs.

**Table S3.** Dielectric strength comparison of common pressure-transmitting medium (PTM).


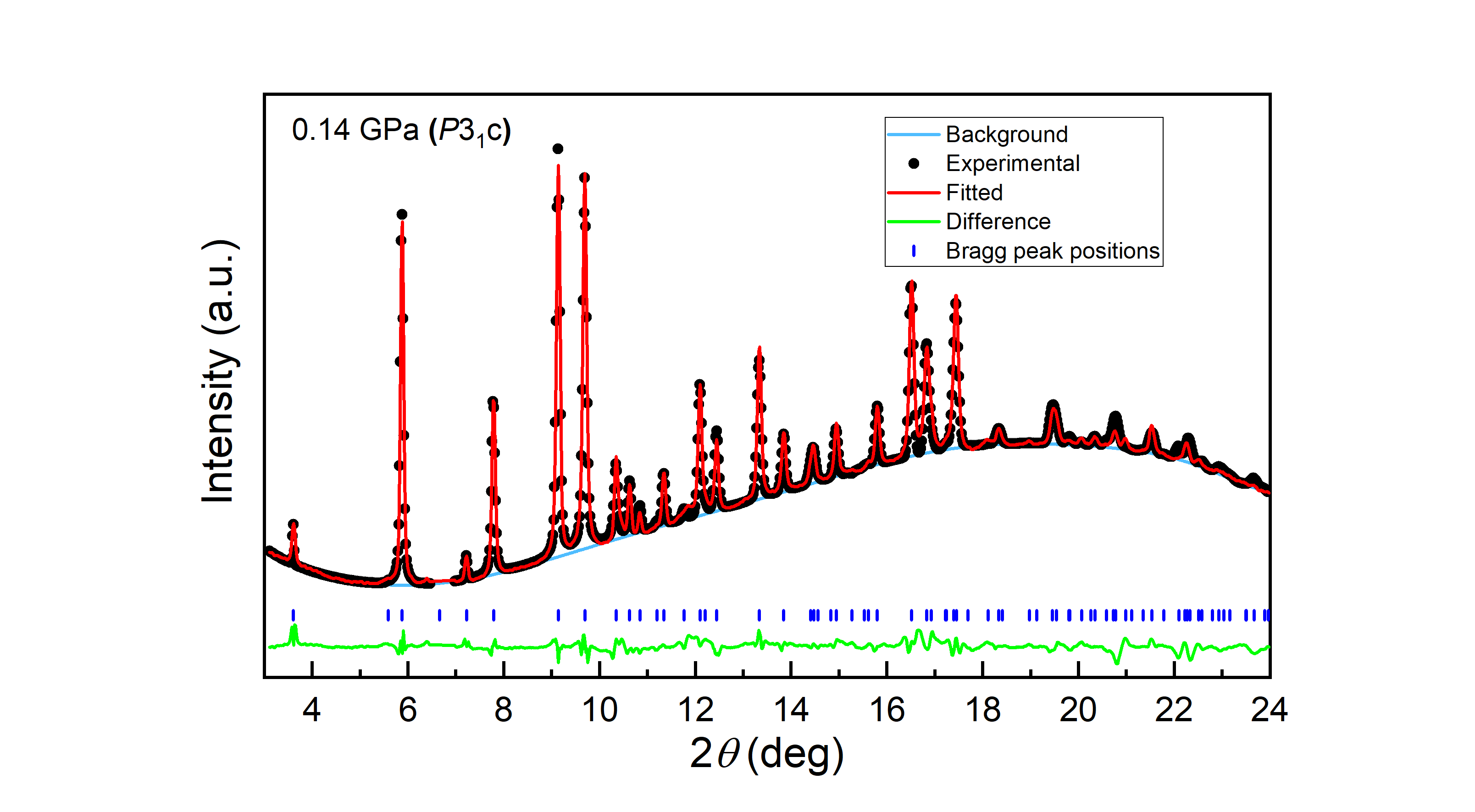
**Figure S1.** Typical Rietveld refinements of BaFe_4_O_7_ at low-pressure phase (1.4 GPa) and the corresponding refined structure


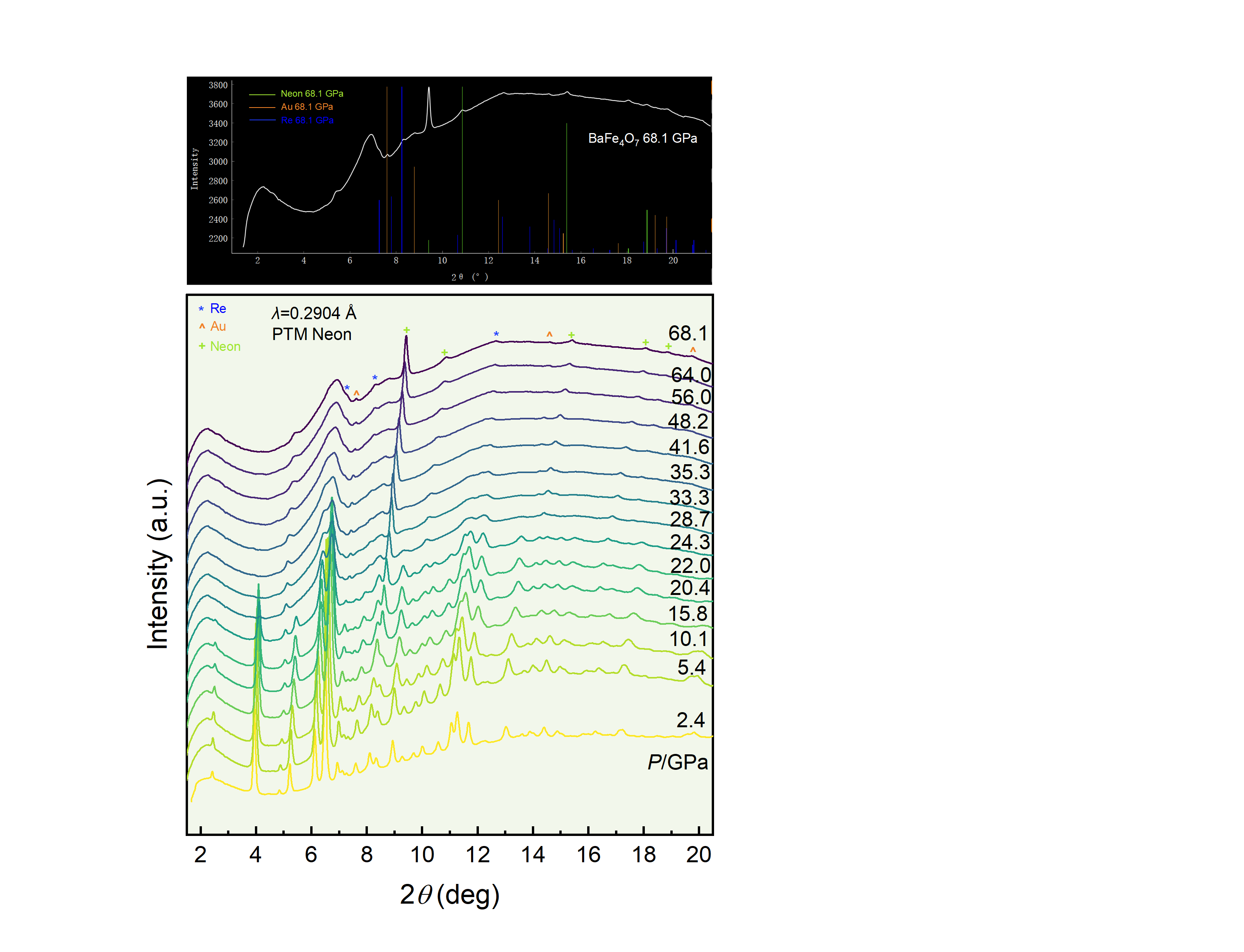
**Figure S2.** Synchrotron P-XRD patterns of BaFe_4_O_7_ with neon as the pressure transmitting medium.


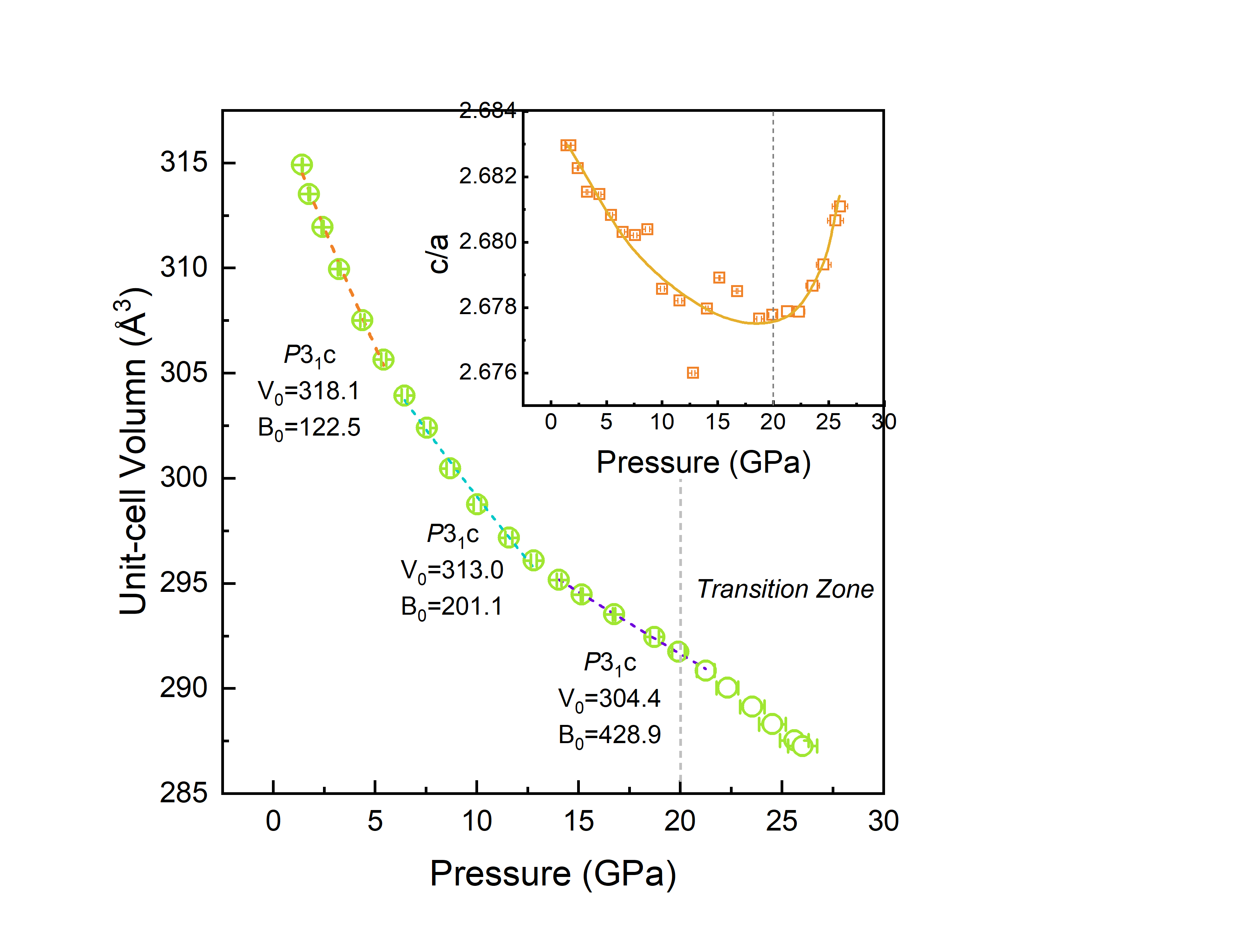


**Figure S3**. Pressure dependence of the unit-cell volume up to 26.0 GPa. The dashed line represents the Birch−Murnaghan third-order equation of state (EoS) fit to the pressure−volume data of the compression below 26.0 GPa. The inset shows the pressure dependence of the c/a ratio up to 26.0 GPa. The colored lines indicate general trends in the data. Pressure error bars are derived from the pressure gradient shown in Figure S12.


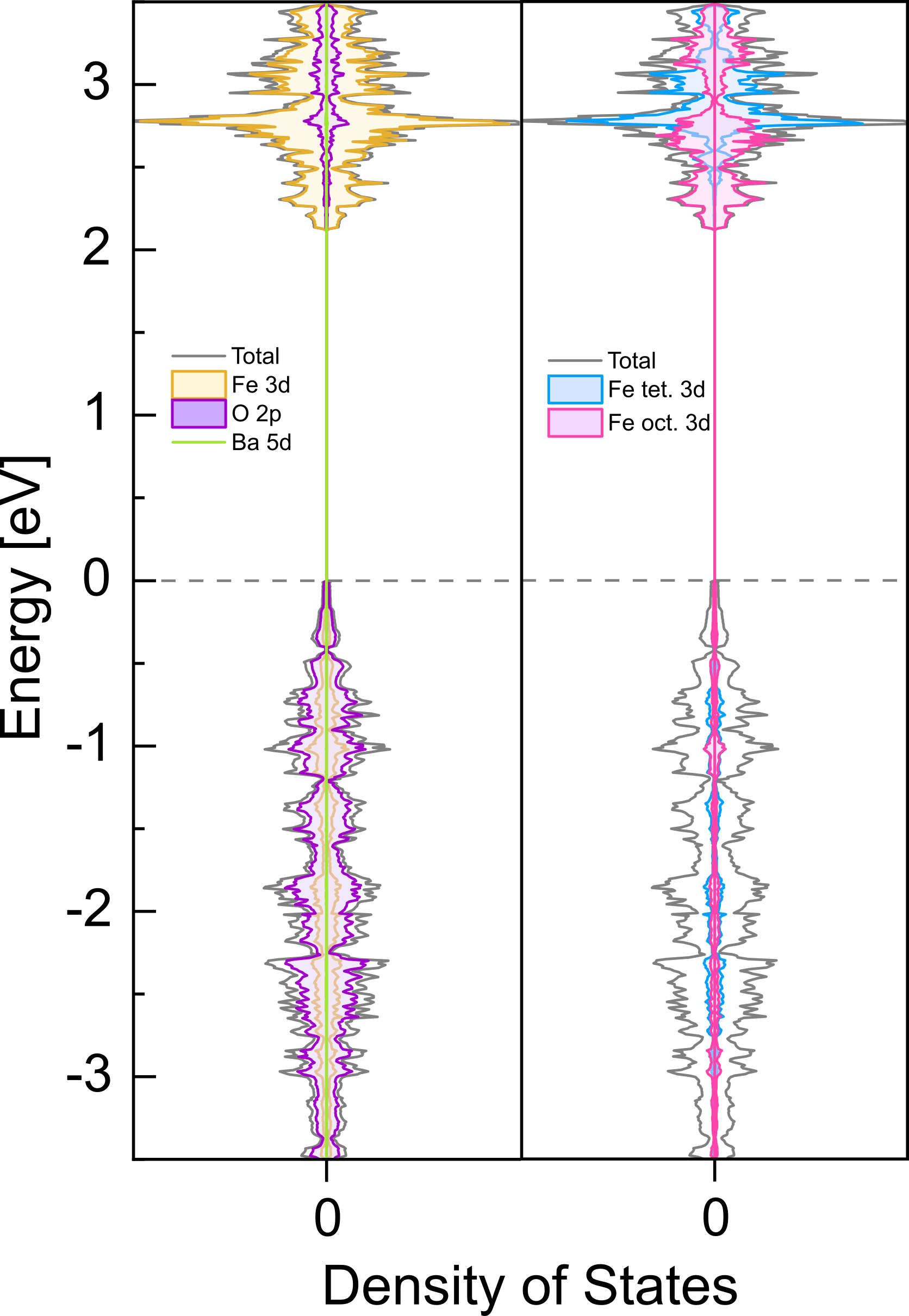
**Figure S4.** Projected density of states (DOS) for BaFe_4_O_7_ at ambient condition.

**
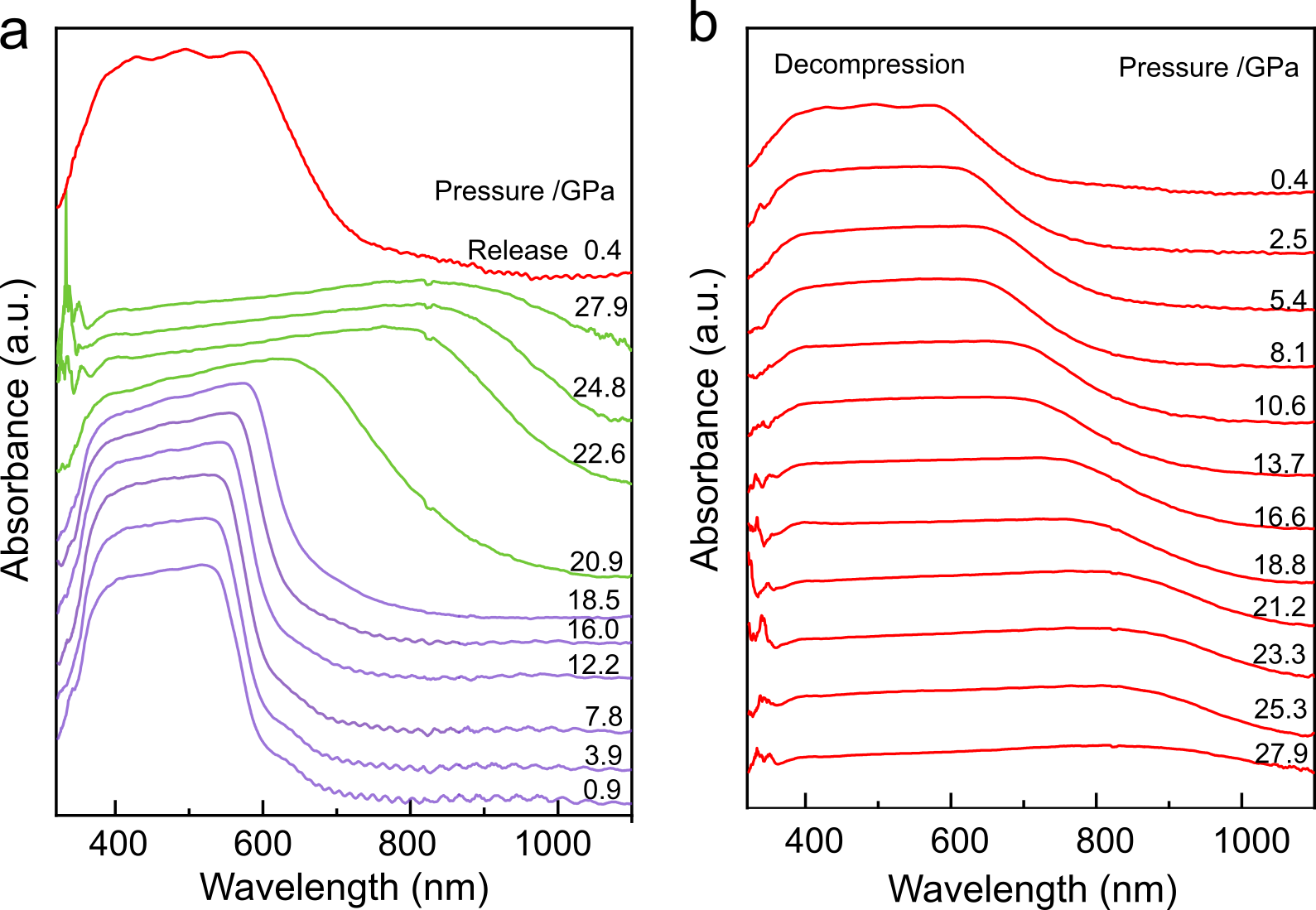
**

**Figure S5.** UV−vis-NIR absorption spectra of BaFe_4_O_7_ at selected pressures during compression (a) and decompression (b) at room temperature.


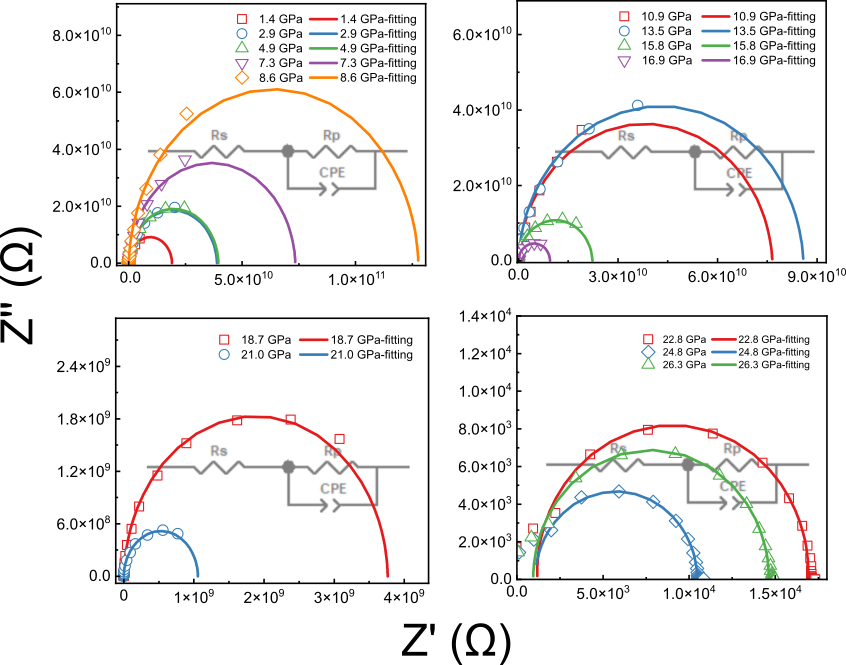


**Figure S6** EIS data (points) and the corresponding fittings(curves) of BaFe_4_O_7_ under high pressure, the insets are the equivalent circuit.


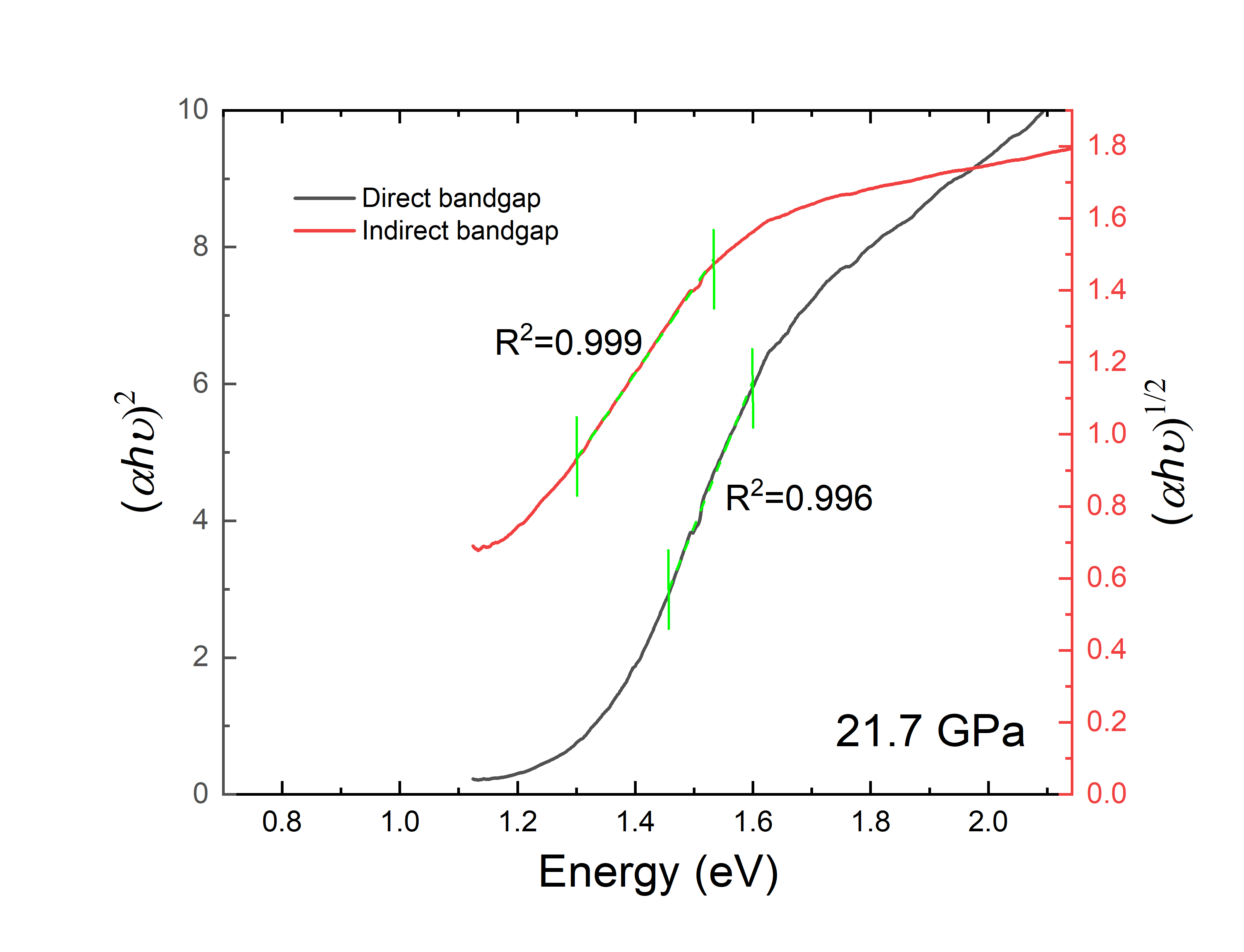


**Figure S7** Tauc plots constructed using direct and indirect transition methods at 21.7 GPa.


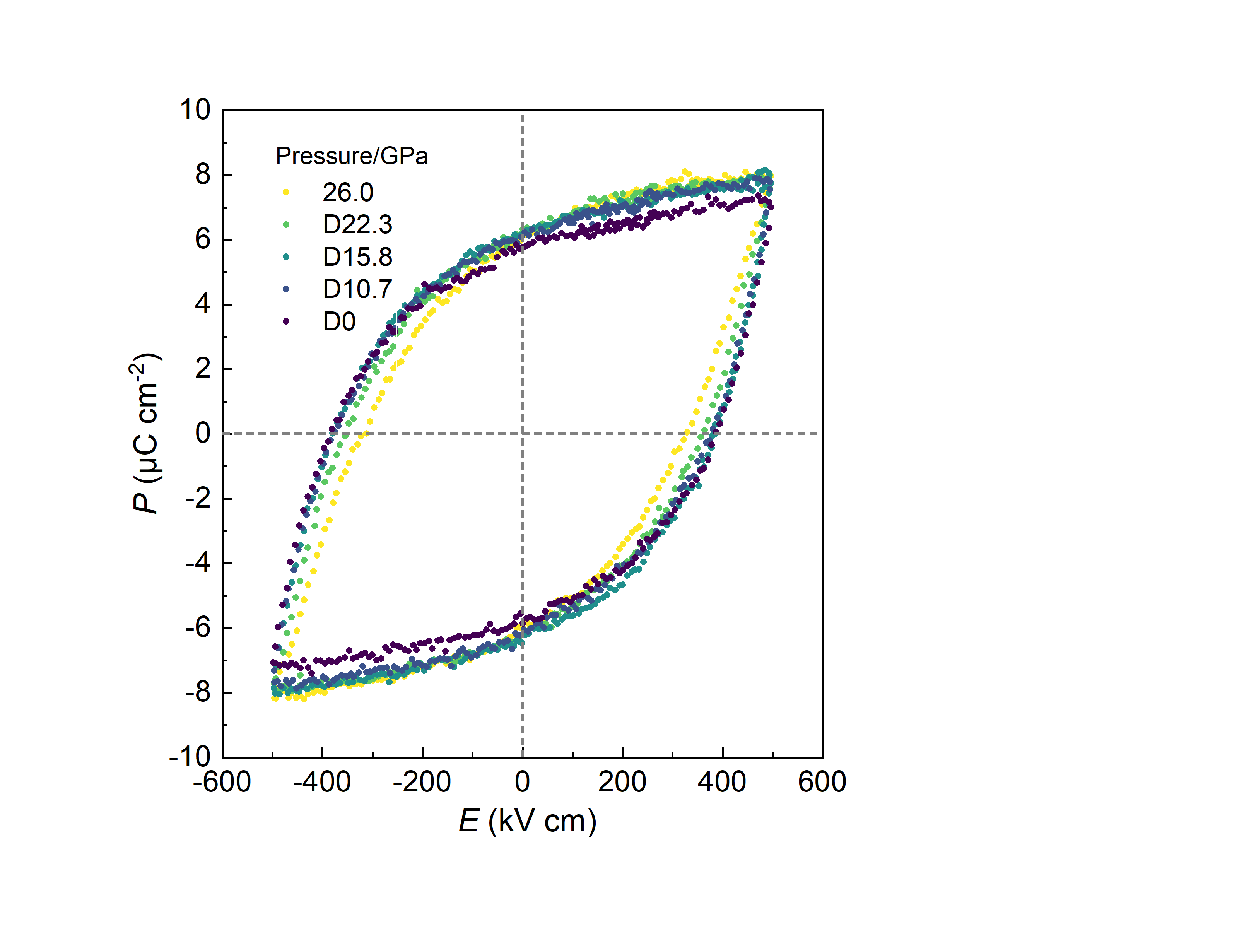
**
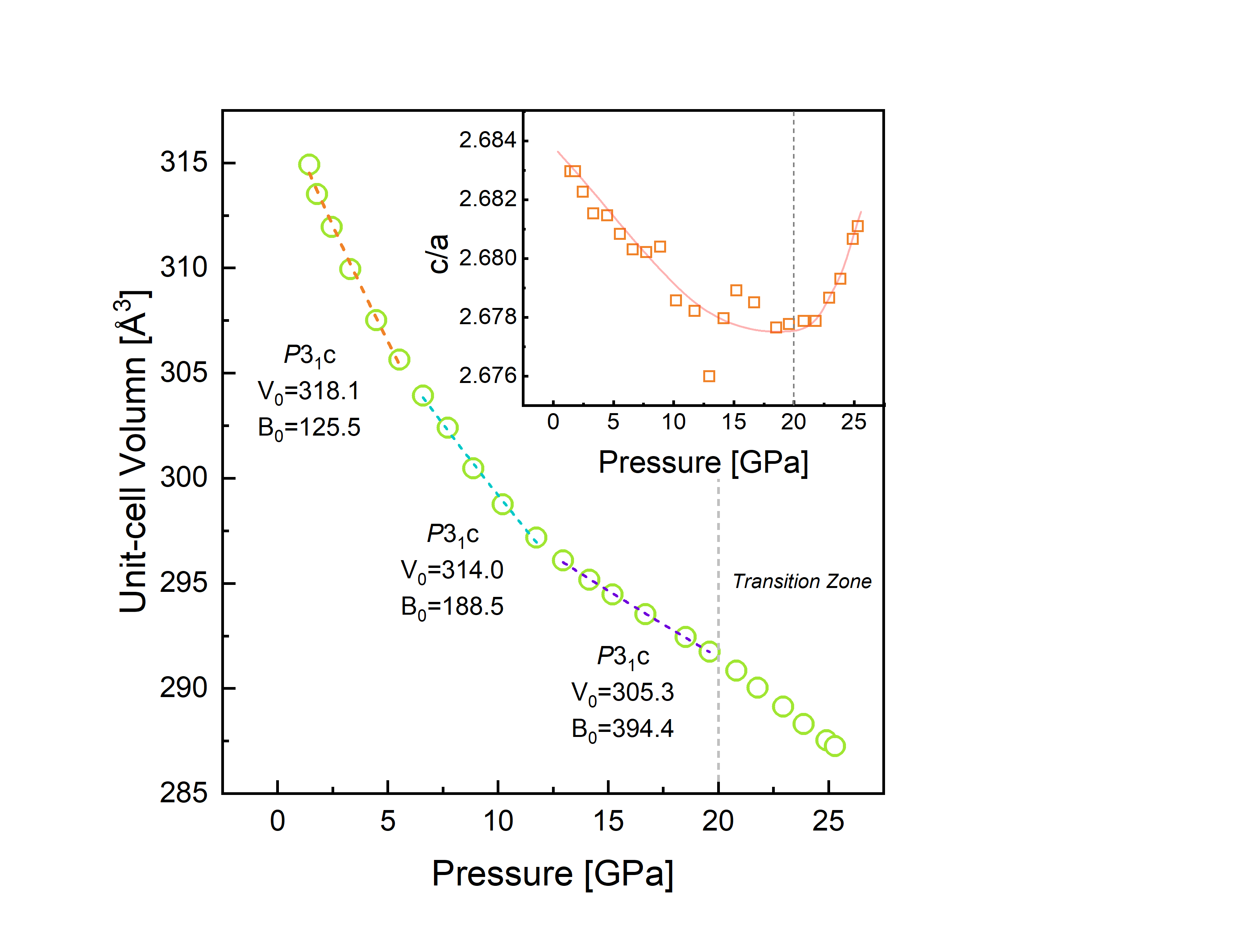
**

**Figure S8**. *P*-*E* hysteresis loops of a ferroelectric capacitor of BaFe_4_O_7_ (10 µm thick) during the decompression process.


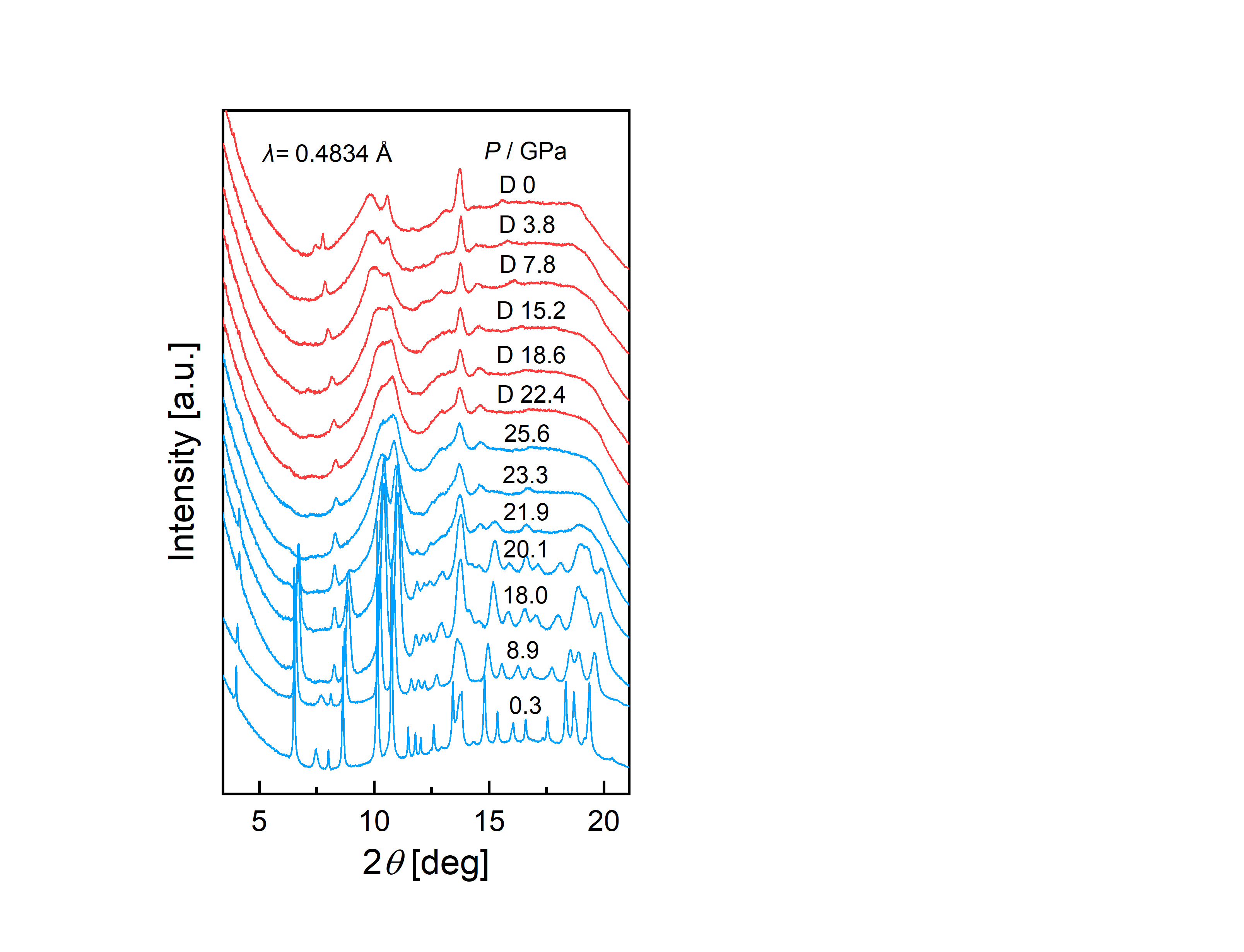


**Figure S9.** Synchrotron P-XRD patterns of BaFe_4_O_7_ during compression and decompression process at room temperature.

**Figure S10** Incident photon-to-current efficiency (IPCE) of BaFe_4_O_7_ under high pressure.
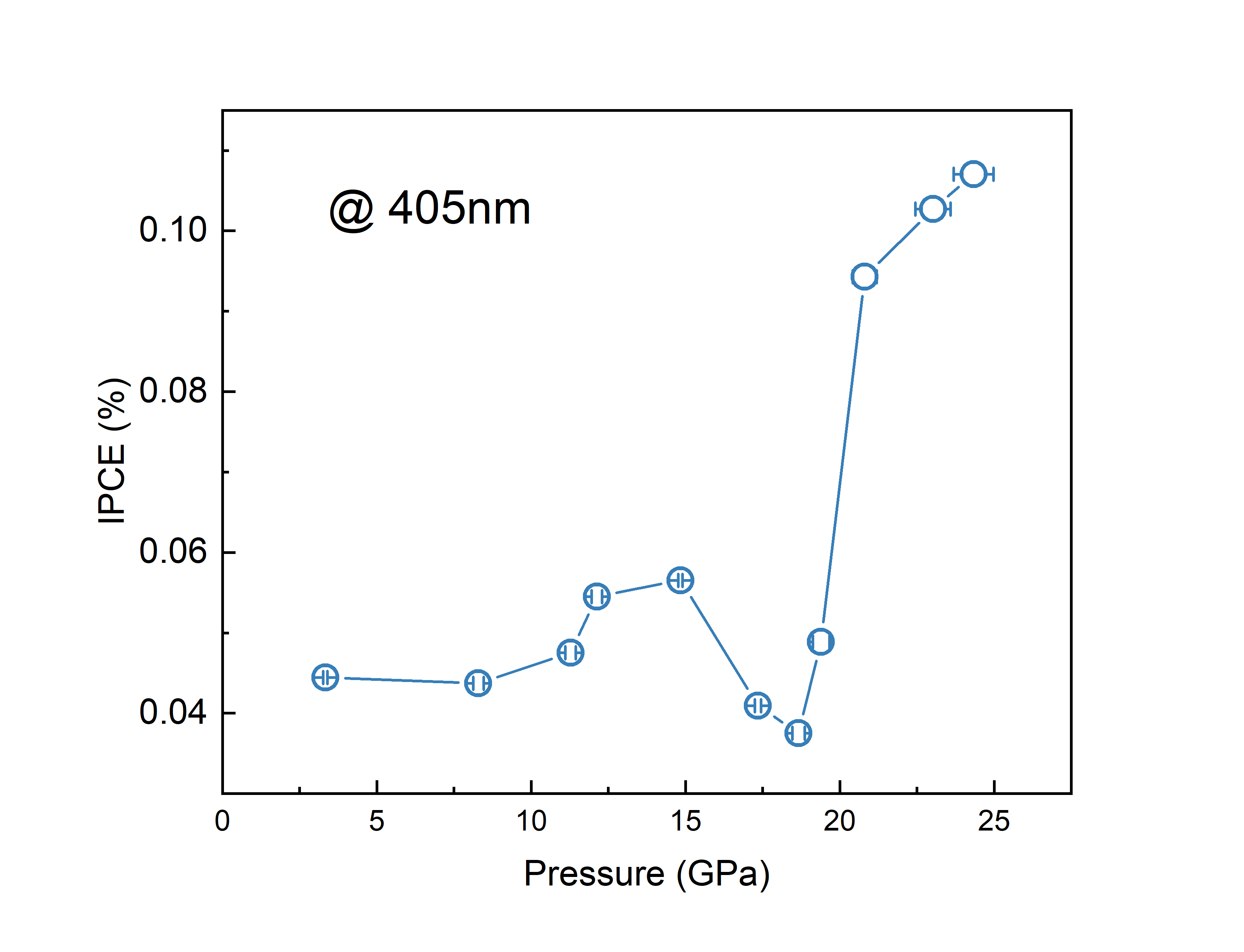


**Figure S1**
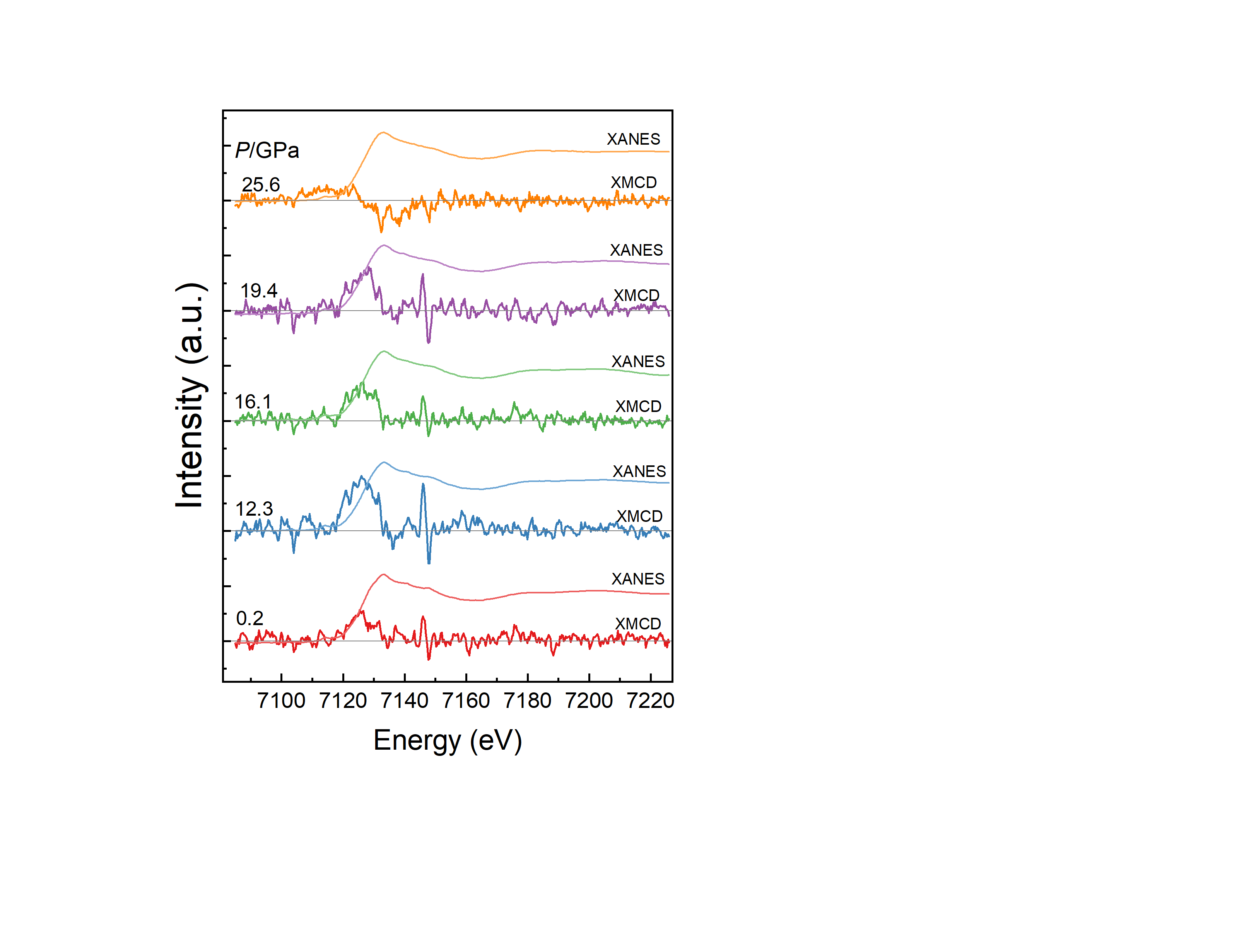
**1** Fe *K*-edge X-ray absorption near edge structure (XANES) and X-ray magnetic circular dichroism (XMCD) spectra at selected pressures at room temperature.

**
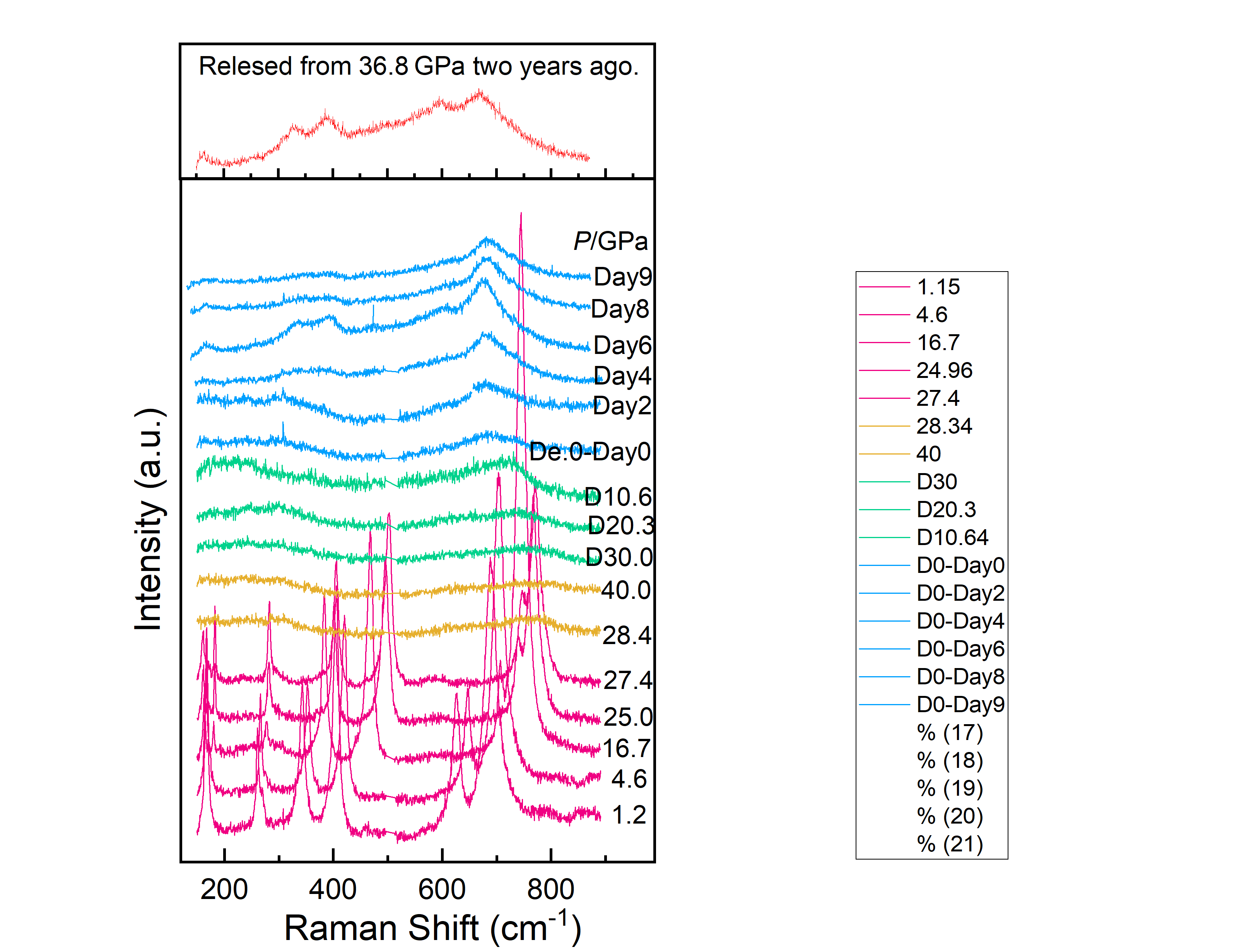
Figure S12** Raman spectra of BaFe_4_O_7_ at selected pressures and time-dependent Raman spectra of the sample released from 40 GPa.


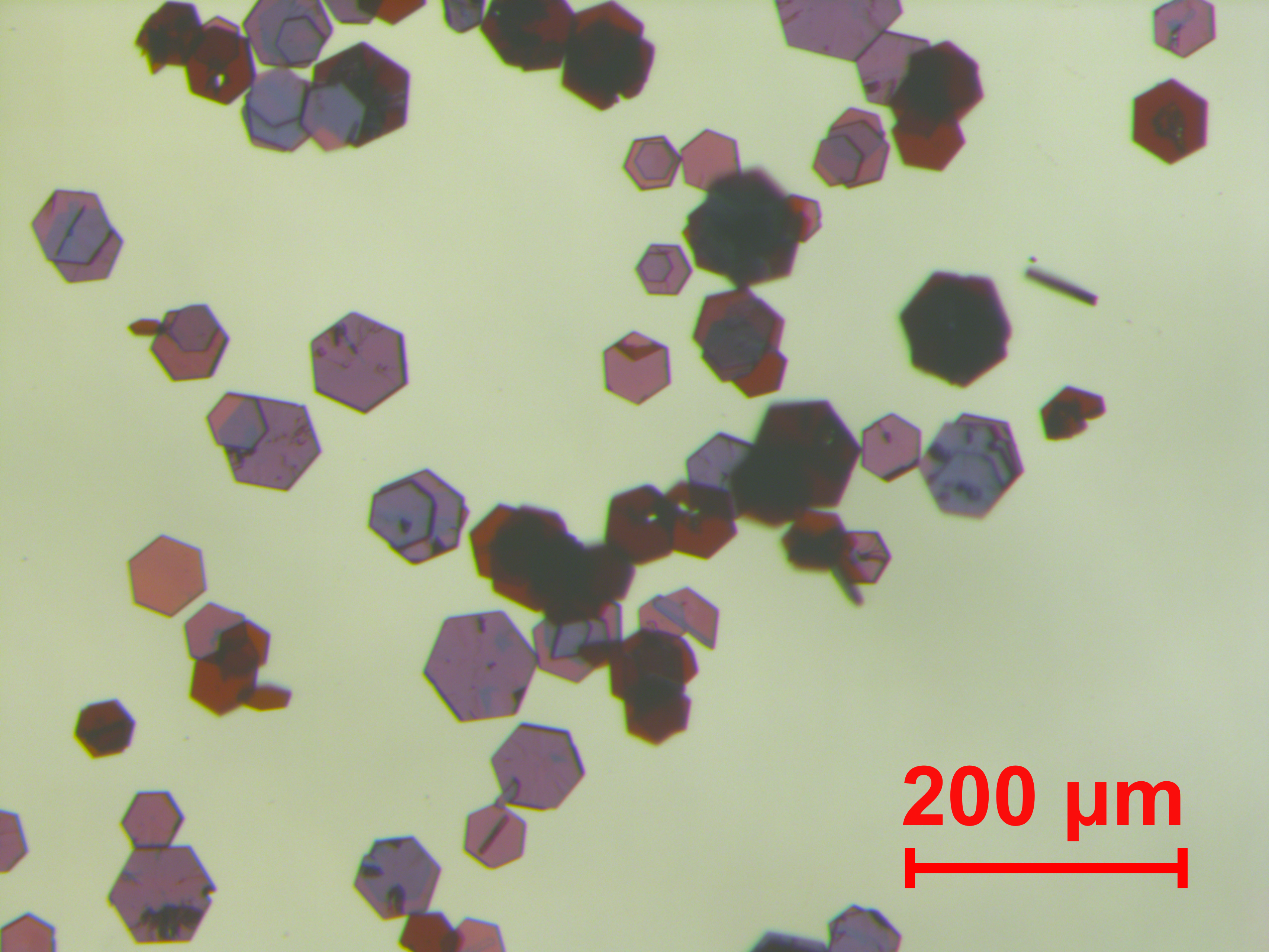
Figure S13 Optical microscopy image of BaFe_4_O_7_.

**Figure S14** The measured XRD pattern at ambient conditions.
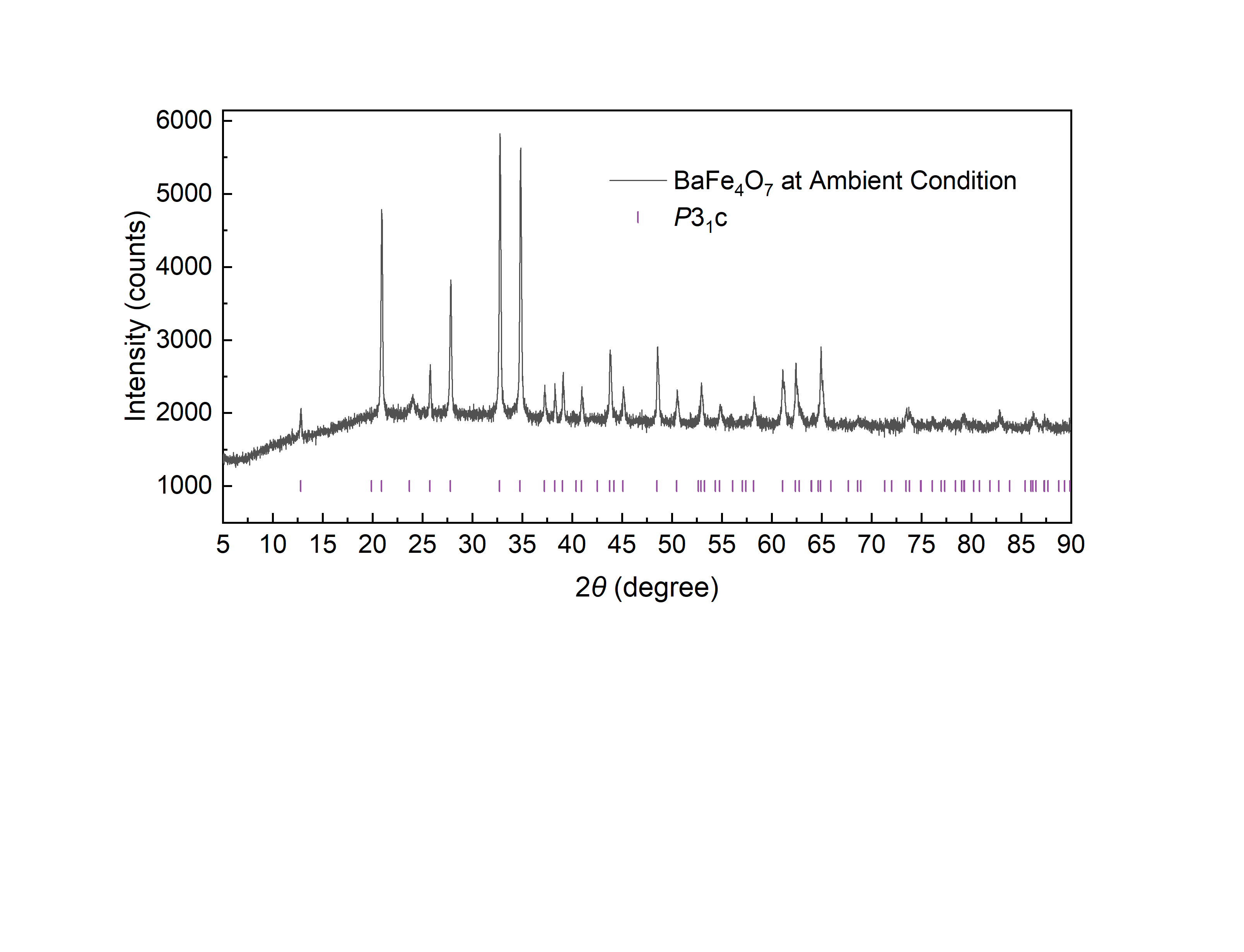


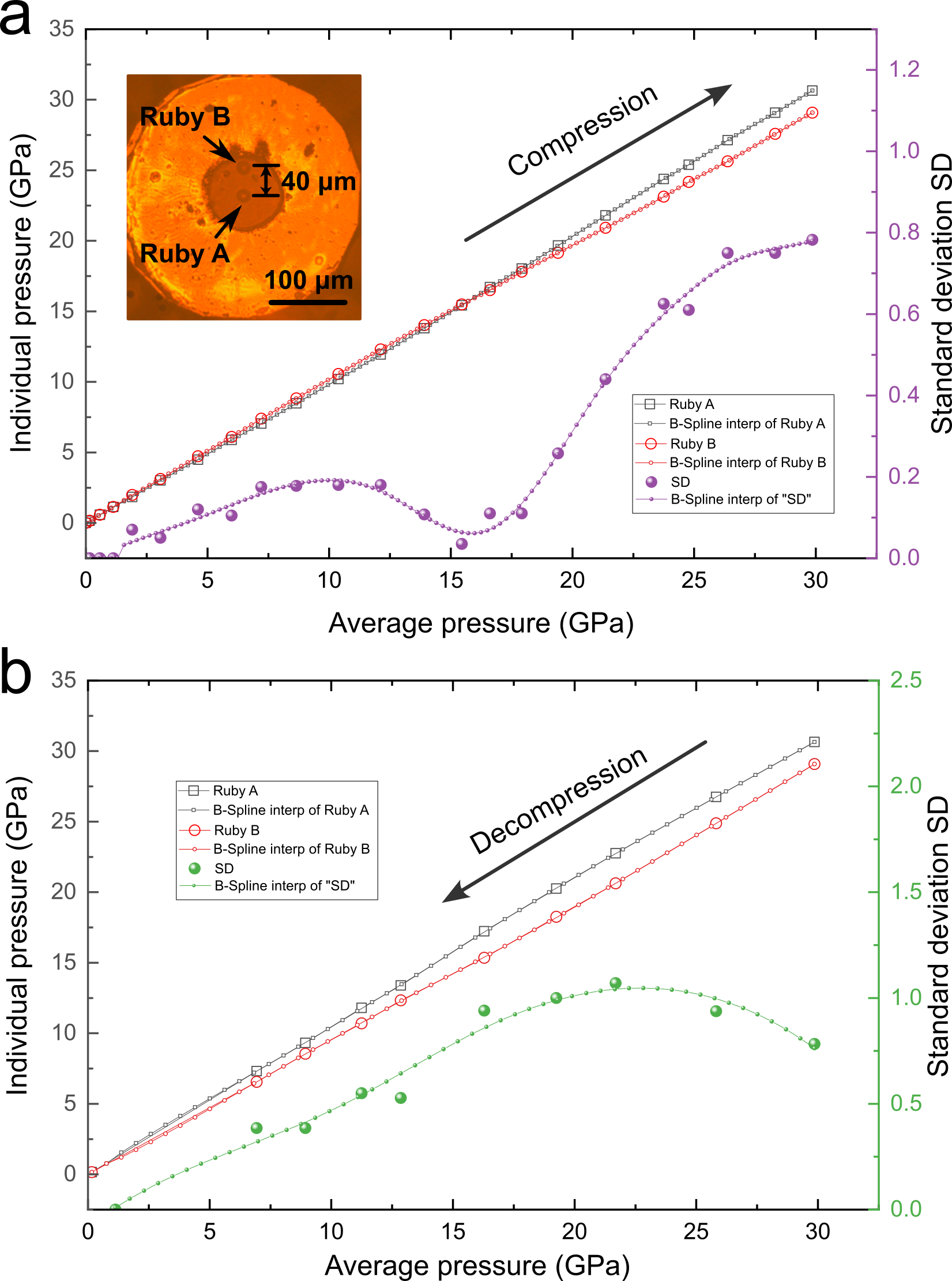
**Figure S15** Pressure dependence of hydrostaticity inside the sample chamber of a diamond anvil cell (DAC) with mineral oil as the PTM upon. a) compression and b) decompression. The hydrostaticity was characterized by the different pressures (open symbols, left vertical axis) obtained from the Ruby A near the center and edge of the sample chamber Ruby B and their standard deviation (solid symbols, right vertical axis). The inset shows an image of the mineral oil at ∼0.5 GPa along with the two ruby spheres inside the DAC.


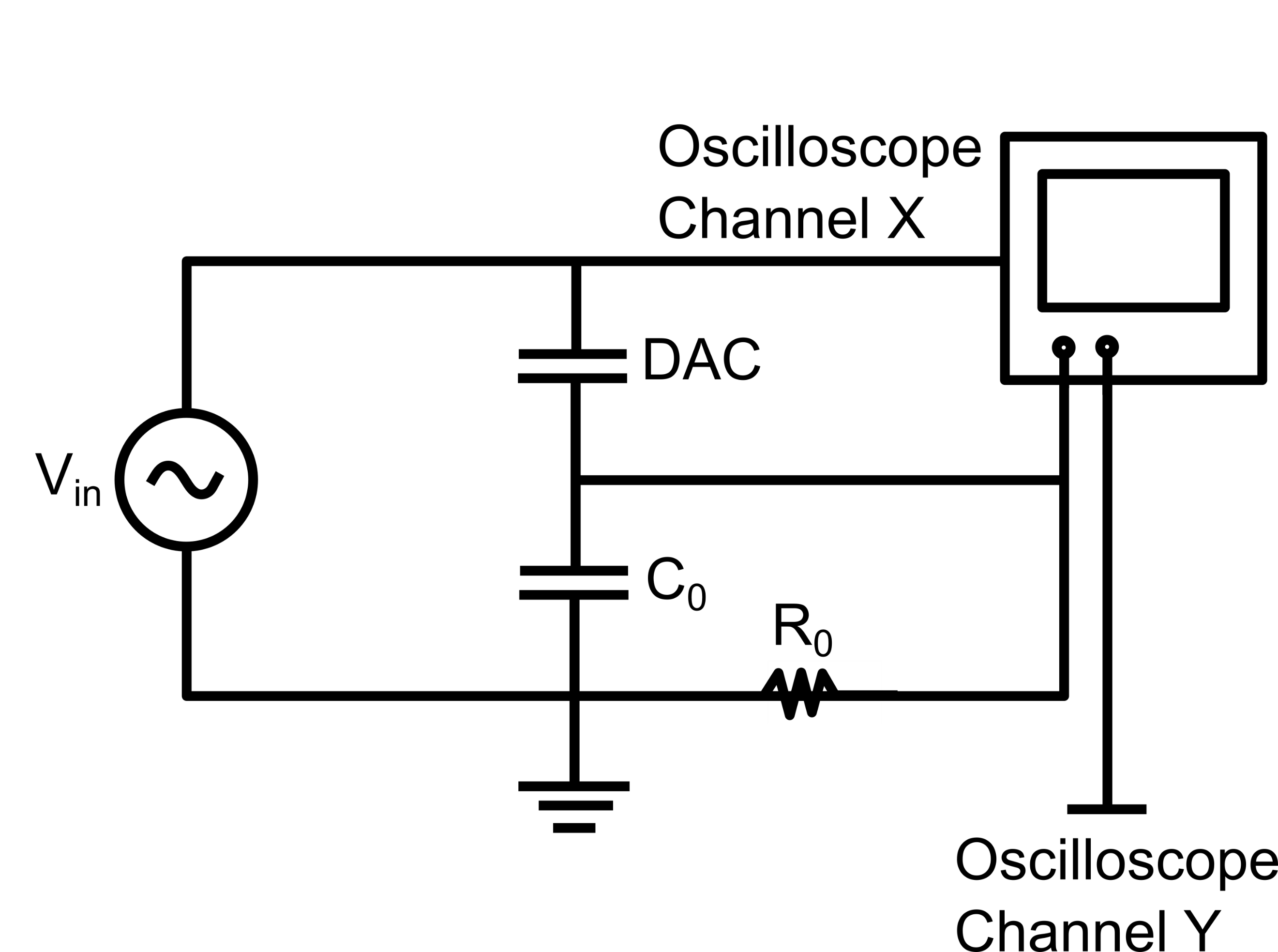


**Figure S16** The circuit diagram for measuring the *P-E* hysteresis loop, where C_0_ is the reference capacitor, and the reference capacitor used in this experiment is 1 nF. DAC is the sample in DAC to be measured, R_0_ is the compensation resistor, which is 20 kΩ in this work. V_in_ is the AC current source, and a triangular wave is used here.

**Table S1.** The Fe-O and Fe-Fe bond length range in ambient BaFe_4_O_7_.

| Bond Type | | Min. Distance  [Å] | Max. Distance  [Å] |
| --- | --- | --- | --- |
| Fe-O | Fe_tet._-O | 1.712 | 2.404 |
|  | Fe_oct._-O | 1.784 | 2.409 |
| Fe-Fe | Fe_oct._- Fe_oct._ | 2.9847 | 2.9856 |
|  | Fe_tet._-Fe | 3.2327 | 3.6836 |

**Table S2.** Literature Data of the bulk PFVs.

| Materials | Materials | *E*_g_  [eV] | *P*_r_  [μC cm^-2^] | Ref. |
| --- | --- | --- | --- | --- |
| Single crystal | BiFeO_3_ | 2.67 | 60 | [1-4] |
|  | BaTiO_3_ | 3.5 | 25 | [5-7] |
|  | LiNbO_3_ | 3.78 | 71 | [8] |
|  | KBiFe_2_O_5_ | 1.61 | 0.39 | [9] |
| Bi-base | (Bi_0.5_Na_0.5_)TiO_3_ (**BNT)** | 2.95 | 32.5 | [10] |
|  | BiFe_0.91_Zr_0.09_O_3_(**BFZO**) | 2.65 | 30.13 | [11] |
|  | Bi_5_FeTi_3_O_15_(**BFTO**) | 2.68 | 0.25 | [12] |
|  | Bi_0.9_La_0.1_FeO_3_(**B0.9L0.1FO**) | 2.8 | 60 | [13] |
|  | Ba_1−x_(Bi_0.5_Li_0.5_)_x_TiO_3_(**BBLT**) | 3.2 | 8 | [14] |
|  | Sr_1-x_Bi_2+x_ Nb_2-x_ Ni_x_ O_9-x_(**SBNN**) | 2.7 | 2.7 | [15] |
|  | Bi_0.85_La_0.15_FeO_3_(**B0.85L0.15FO**) | 2.63 | 17 | [16] |
|  | Ba_0.5_(Bi_0.5_K_0.5_)_1.5_TiO_3_(**BBKT**) | 2.62 | 4.45 | [17] |
| Pb-base | Pb(Zr,Ti)O_3_(**PZT**) | 3.6 | 7 | [18] |
|  | (Pb_0.97_La_0.03_)(Zr_0.52_Ti_0.48_)O_3_(**PLZT3/52/48**) | 3.4 | 45 | [19, 20] |
| Solid solutions | 0.3Bi_0.5_Na_0.5_TiO_3_-0.7BiFeO_3_(**0.3BNT-0.7BFO**) | 1.83 | 4.64 | [10] |
|  | Pb(In_1/2_Nb_1/2_))O_3_–Pb(Mg_1/3_Nb_2/3_))O_3_–PbTiO_3_(**PIMN–PT**) | 2.67 | 30 | [21] |
|  | 0.8NaNbO_3_-0.2La(Mn_0.5_Ni_0.5_)O_3_(**NLMNO**) | 2.21 | 1 | [22] |
|  | 0.5PbTiO_3_-0.5Bi(Ni_0.5_Ti_0.5_)O_3_(**0.5PT-0.5BNT**) | 2.2 | 38 | [23] |
|  | 0.65PbTiO_3_-0.35Bi(Ni_2/3_+xNb_1/3−x_)O_3−δ_(**PT-BNN**) | 2.9 | 33 | [24] |
|  | (1-x)(Na_0.5_K_0.5_)NbO_3_-xBa(Ni_0.5_Nb_0.5_)O_3−δ_(**KNBNN**) | 1.63 | 11 | [25] |
|  | 0.9BaTiO_3_-0.1Ba(Ni_0.5_Nb_0.5_)O_3−δ_(**BTNN**) | 1.5 | 4 | [26] |
|  | PbTiO_3_-BiFeO_3_-Bi(Ni_0.5_Ti_0.5_)O_3_(**PT−BF−BNT**) | 2.85 | 39 | [23] |
| Others | (KNbO_3_)_0.9_ (BaNi_0.5_Nb_0.5_O_3_)_0.1_(**KBNNO**) | 1.39 | 1.4 | [27] |
|  | NaNbO_3_ | 3.42 | 35 | [22] |

**Table S3**: Dielectric strength comparison of common PTMs.

| PTM | Dielectric Strength (kV/mm) |
| --- | --- |
| Mineral oil (liquid) | 10-15 |
| Silicone oil (liquid) | 12-15 |
| Neon (gas) | ~ 1 |
| Argon (gas) | ~ 1.2-1.5 |
| Helium (gas) | ~ 0.7-1 |

1. J. Wang, J. B. Neaton, H. Zheng, V. Nagarajan, S. B. Ogale, B. Liu, D. Viehland, V. Vaithyanathan, D. G. Schlom, U. V. Waghmare, N. A. Spaldin, K. M. Rabe, M. Wuttig, R. Ramesh. *Science*, **2003**, 299, 1719-1722.
2. H. T. Yi, T. Choi, S. G. Choi, Y. S. Oh, S. W. Cheong. *Adv. Mater.* **2011**, 23, 3403-3407.
3. X. S. Xu, T. V. Brinzari, S. Lee, Y. H. Chu, L. W. Martin, A. Kumar, S. McGill, R. C. Rai1, R. Ramesh, V. Gopalan, S. W. Cheong, J. L. Musfeldt. *Phys. Rev. B*, 2009, 79, 134425.
4. J. F. Ihlefeld, N. J. Podraza, Z. K. Liu, R. C. Rai, X. Xu, T. Heeg, Y. B. Chen, J. Li, R. W. Collins, J. L. Musfeldt, X. Q. Pan, J. Schubert, R. Ramesh, D. G. Schlom. *Appl. Phys. Lett.* **2008**, 92, 142908.
5. Z. Gu, D. Imbrenda, A. L. Bennett-Jackson, M. Falmbigl, A. Podpirka, T. C. Parker, D. Shreiber, M. P. Ivill, V. M. Fridkin, J. E. Spanier. *Phys. Rev. Lett.* **2017** 118, 096601.
6. A. M. Burger, R. Agarwal, A. Aprelev, E. Schruba, A. Gutierrez-Perez, V. M. Fridkin, J. E. Spanier. *Sci. Adv.* **2019**, 5,eaau5588.
7. F. Liu, I. Fina, D. Gutiérrez, G. Radaelli, R. Bertacco, J. Fontcuberta. *Adv. Electron. Mater.* 2015, 1 1500171.
8. R. S. Weis, T. K. Gaylord. *Appl. Phys. A* **1985**, 37, 191-203.
9. G. Zhang, H. Wu, G. Li, Q. Huang, C. Yang, F. Huang, F. Liao, J. Lin. *Sci. Rep.* **2013**, 3, 1265.
10. Y. Gong, C. Chen, F. Zhang, X. He, H. Zeng, Q. Yang, Y. Li, Z. Yi. *J Am Ceram Soc.* **2020**, 103, 4363-4372.
11. K. Chandrakanta, R. Jena, P. Pal, Md.F. Abdullah, S.D. Kaushik, A.K. Singh. *Int. J. Miner. Metall. Mater3* **2021**,28, 1861-1867.
12. Z. Fan, W. Ji, T. Li, J. Xiao, P. Yang, K. P. Ong, K. Zeng, K. Yao, J. Wang. *Acta Mater.* **2015**, 88 ,83-90.
13. Q. Zhang, F. Xu, M. Xu, L Li, Y. Lu, M. Li, P. Li, M. Li, G. Chang, Y. He. *Mater. Res. Bull.* **2017**, 95, 56-60.
14. S. Pal, A. B. Swain, P. P. Biswas, D. Murali, A. Pal, B. R. K. Nanda, Pattukkannu. Murugavel. *Sci. Rep.* **2018**, 8, 8005.
15. M. Wu, X. Lou, T. Li, J. Li, S. Wang, W Li, B Peng, G. Gou. *J. Alloys Compd.* **2017**, 724, 15, 1093-1100.
16. J. Chen, W. Pei, G. Chen, Q. Zhang, Y. Lu, H. Huang, M. Li, Y. He. *J Am Ceram Soc.* **2018**, 101, 4892-4898.
17. S. Pal, S. Muthukrishnan, B. Sadhukhan, S. N. V., D. Murali, P. Murugavel. *J. Appl. Phys.* **2021**, 129, 084106.
18. J. F. Scott. *Jpn. J. Appl. Phys.* **1999**, 38, 2272.
19. J. Zhang, X. Su, M. Shen, Z. Dai, L. Zhang, X. He, W. Cheng, M. Cao, G. Zou. *Sci. Rep.* 2013, 3, 2109.
20. K. Uchino, Y. Miyazawa, S. Nomura. *Jpn. J. Appl. Phys.***1983**, 22, 102.
21. X. Qi, K. Li, E. Sun, B. Song, D. Huo, J. Li, X. Wang, R. Zhang, B. Yang, W. Cao. *Chin. J. Met. Sci. Technol.* **2022**, 104, 30, 119-126.
22. J. Wang, Z. Peng, J. Wang, D. Wu, Z. Yang, X. Chao. *Scr. Mater.* **2022**, 221, 114976.
23. L. Wu, A. M. Burger, A. L. Bennett-Jackson, J. E. Spanier, P. K. Davies. *Adv. Electron. Mater*. **2021**, 7, 2100144.
24. H. Liu, J. Chen, Y. Ren, L. Zhang, Z. Pan, L. Fan, X. Xing. *Adv. Electron. Mater.* **2015**, 1, 1400051.
25. Y. Bai, P. Tofel, J. Palosaari, H. Jantunen, J. Juuti. *Adv. Mater.* **2017**, 29, 1700767.
26. L. Wu, A. R. Akbashev, A. A. Podpirka, J. E. Spanier, P. K. Davies. *J. Am. Ceram. Soc.* **2019**, 102, 4188-4199.
27. E. Mainimo, G. W. Ejuh, J. M. B. Ndjaka. *J. Mater. Sci: Mater. Electron* **2020**, 31, 21923-21933.
